# SRPK2 Mediates HBV Core Protein Phosphorylation and Capsid Assembly via Docking Interaction

**DOI:** 10.1371/journal.ppat.1011978

**Published:** 2024-02-07

**Authors:** Ryan Pak Hong YIP, Doris Ching Ying Kwok, Louis Tung Faat Lai, Siu-Ming Ho, Ivan Chun Kit Wong, Chi-Ping Chan, Wilson Chun Yu Lau, Jacky Chi Ki Ngo

**Affiliations:** 1 School of Life Sciences, The Chinese University of Hong Kong, Shatin, N.T., Hong Kong SAR, China; 2 School of Biomedical Sciences, The University of Hong Kong, Pokfulam, Hong Kong, Hong Kong SAR, China; 3 State Key Laboratory of Liver Research (The University of Hong Kong), Pokfulam, Hong Kong, Hong Kong SAR, China; 4 Center for Soybean Research of the State Key Laboratory of Agrobiotechnology, The Chinese University of Hong Kong, Shatin N.T., Hong Kong SAR, China; 5 Center for Novel Biomaterials, The Chinese University of Hong Kong, Shatin N.T., Hong Kong SAR, China; 6 Center for Protein Science and Crystallography, The Chinese University of Hong Kong, Shatin N.T., Hong Kong SAR, China; Pennsylvania State University College of Medicine: Penn State College of Medicine, UNITED STATES

## Abstract

Members of the serine–arginine protein kinase (SRPK) family, SRPK1 and SRPK2, phosphorylate the hepatitis B core protein (Cp) and are crucial for pregenomic RNA encapsidation during viral nucleocapsid assembly. Among them, SRPK2 exhibits higher kinase activity toward Cp. In this study, we identified Cp sites that are phosphorylated by SRPK2 and demonstrated that the kinase utilizes an SRPK-specific docking groove to interact with and regulate the phosphorylation of the C-terminal arginine rich domain of Cp. We determined that direct interaction between the docking groove of SRPK2 and unphosphorylated Cp inhibited premature viral capsid assembly *in vitro*, whereas the phosphorylation of the viral protein reactivated the process. Pull-down assays together with the new cryo-electron microscopy structure of the HBV capsid in complex with SRPK2 revealed that the kinases decorate the surface of the viral capsid by interacting with the C-terminal domain of Cp, underscoring the importance of the docking interaction in regulating capsid assembly and pregenome packaging. Moreover, SRPK2-knockout in HepG2 cells suppressed Cp phosphorylation, indicating that SRPK2 is an important cellular kinase for HBV life cycle.

## Author summary

Hepatitis B virus (HBV) is an enveloped DNA virus that causes hepatitis B, a common liver infection. HBV infection is highly associated with the development of both acute and chronic liver diseases, ranging from cirrhosis to liver cancer and liver failure. Like many viruses, HBV does not possess adequate endogenous factors for self-replication. Thus, it relies on host metabolism and machinery. Members of the SRPK family have been identified to the host cellular kinases that interact with HBV core protein during viral core assembly. Here we report the mechanism of how SRPK2 interacts with and phosphorylates HBV core protein. We also highlight the role of SRPK2 in the phosphorylation of HBV core protein in cells. Our research laid the foundation to targeting this host cellular kinase and developing new means to combat HBV infection.

## Introduction

Chronic hepatitis B infection is a global health problem. In 2019, an estimated 316 million individuals were infected with hepatitis B virus (HBV) and 555,000 individuals died of HBV-related causes worldwide [1]. Moreover, HBV infects 1.5 million new individuals each year. HBV, which belongs to the Hepadnaviridae family, is a pathogen that causes acute and chronic hepatitis. Chronic HBV infection is highly related to severe liver diseases and contributes to 80% of primary hepatocellular carcinoma globally [2]. Current treatments, such as immunomodulators and antivirals, have their drawbacks [3–5]. Thus, new therapeutic strategies and targets are required.

The HBV replication cycle has been largely studied [6–12]. Nucleocapsid formation is a substantial event in this cycle. During nucleocapsid formation, newly translated hepatitis B core protein (Cp) recognizes and encapsidates the pregenomic RNA/reverse transcriptase (pgRNA/RT) complex. The reverse transcription of pgRNA into relaxed circular DNA (rcDNA) results in the maturation of nucleocapsids. These nucleocapsids are then either reimported to the nucleus to enhance the covalently closed circular DNA (cccDNA) pool or enveloped in the endoplasmic reticulum lumen and secreted as virions.

Cp is an elementary component of nucleocapsids and normally exists as a homodimer [13,14]. It consists of an N-terminal assembly domain (residues 1–149) and a C-terminal arginine-rich domain (ARD, residues 150–185) [15]. Truncated Cp containing only the assembly domain can lead to the self-assembly of viral capsids. The ARD interacts with RNA and is vital for the encapsidation of viral pgRNA. In addition, the ARD enhances the stability of the assembled nucleocapsid after the encapsidation of pgRNA [16,17].

Phosphorylation is not required for capsid assembly whereas unphosphorylated Cp alone tends to self-assemble into empty capsids, an icosahedral core containing 240 copies of core protein, *in vitro*. However, prior to capsid assembly, Cp undergoes phosphorylation, which is crucial for the encapsidation of the viral pregenome and formation of the nucleocapsid [18,19]. Among seven sites in the ARD undergo phosphorylation, three serine residues (S157, S164, and S172) are considered major phospho-acceptors, whereas the other four residues (T162, S170, S178, and S180) are residual phosphorylation sites [20–22]. S164 and S172 substantially contribute to pgRNA packaging [20,23], whereas the other sites exert their effects at different stages of the HBV life cycle [18,24].

Members of the serine/arginine-rich (SR) protein kinase (SRPK) family, SRPK 1 and 2, have been identified as major host cellular kinases for the phosphorylation of Cp during HBV replication [21]. SRPKs are crucial for pre-mRNA splicing, and they specifically recognize and phosphorylate serine residues in the arginine/serine (RS) dipeptide within the RS-rich domain of their substrates. SRPK1 and SRPK2 are highly homologous in their kinase core domains, and both kinases possess a conserved electronegative docking groove that regulates the binding and phosphorylation mechanisms of diverse substrates [25–28]. Although the role of the docking groove in the binding and phosphorylation of Cp remains unclear, Zlotnick and colleagues elucidated that SRPK1 acts as a chaperone to gate capsid assembly. By binding to the ARD and forming a complex with Cp, SRPK1 prevents self-assembly of capsid prior to the phosphorylation event [15]. Such gating mechanism may minimize the premature self-assembly and packaging of nonspecific RNA. Given the importance of the SRPK-specific docking groove, we hypothesize that this docking groove governs the gating mechanism.

In this study, we elucidated the molecular mechanisms underlying the binding and phosphorylation of Cp by SRPK2 and determined the critical roles of the docking groove. We examined the cryo-electron microscopy (cryo-EM) structure of the SRPK2/CpWT capsid complex and determined that similar to SRPK1, SRPK2 was bound to identical sites on the capsid and gated the assembly in a phosphorylation-dependent manner. In addition, phosphorylation of Cp in HepG2 cells was suppressed upon the knockout of SRPK2. Together, our findings provide insights into how SRPK2 regulates the phosphorylation of Cp and potentially capsid assembly in HBV life cycle.

## Results

### Interactions between SRPKs and Cp

**Fig 1:**
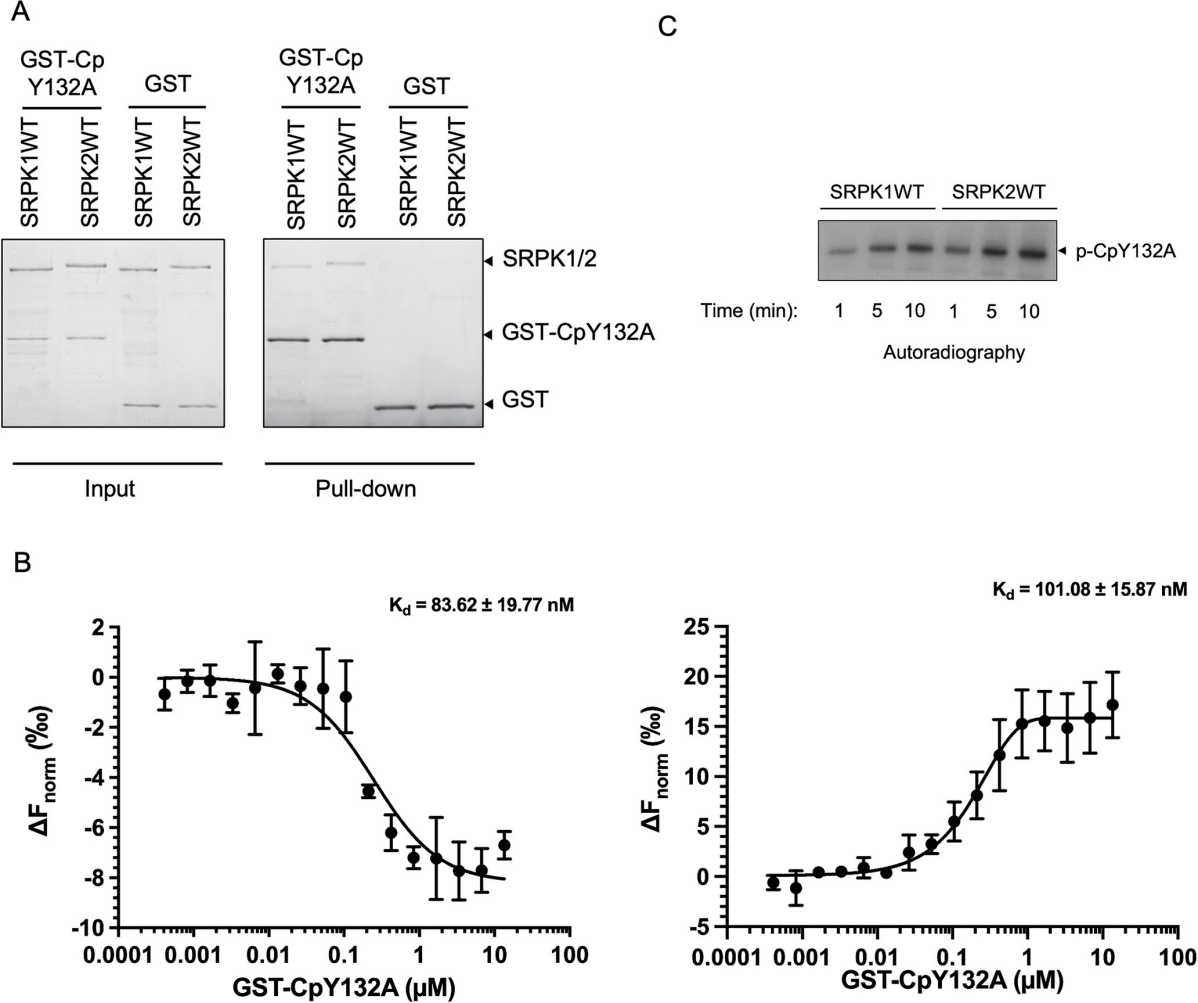
Interactions of SRPK1 and SRPK2 with Cp. (A) *In vitro* GST pull-down assay was performed using recombinant GST-tagged CpY132A with His-tagged SRPK1WT or SRPK2WT. The result was analyzed by SDS-PAGE. Both SRPK1 and SRPK2 bind Cp directly. (B) Microscale thermophoresis (MST) analysis to measure the binding affinity between GST-CpY132A and SRPK1WT or SRPK2WT. K_d_ of 83.6 ± 19.8 nM and 101.1 ± 15.9 nM were determined for the interactions of GST-CpY132A with SRPK1WT (left panel) and SRPK2 WT (right panel), respectively. Values are mean ± S.D. for three independent experiments. (C) *In vitro* radioactive phosphorylation assays of purified CpY132A. Recombinant GST-CpY132A was phosphorylated by SRPK1 or SRPK2 using [^32^P]ATP. Reactions were quenched by SDS loading buffer at the indicated time points and the reactions were resolved by SDS-PAGE followed by autoradiography. SRPK2 phosphorylated Cp more effectively than SRPK1 under the same condition. Experiments have been repeated three times and the representative data are shown.

SRPK1 and SRPK2 are major cellular kinases that phosphorylate Cp *in vivo* [21]. The findings of interaction and activity assays performed using transiently overexpressed kinases indicated that SRPK2 exhibited a higher binding affinity and kinase activity toward Cp than did SRPK1. To determine whether the observed effect is due to a direct interaction between SRPKs and Cp, we examined how SRPK1 and SRPK2 bind and phosphorylate Cp *in vitro* by using recombinant proteins. Recombinant Cp containing the mutation Y132A (hereinafter referred to as the CpY132A), which maintains Cp in its dimeric form and prevents capsid formation, was fused to a GST tag for pull-down study [29]. The results of the GST pull-down assay revealed that both SRPK1 and SRPK2 bind to GST–CpY132A in a similar manner (Fig 1A). Microscale thermophoresis was performed to analyze the binding affinities of SRPK1 and SRPK2 with CpY132A (Fig 1B). The K_d_ values of SRPK1 and SRPK2 with the CpY132A were 83.6 ± 19.8 and 101.1 ± 15.9 nM, respectively, indicating that they bind to Cp with comparable affinities. In addition, we compared the activities of SRPK1 and SRPK2 toward the CpY132A and determined that SRPK2 exhibited slightly higher phosphorylation activity (Fig 1C).

### Docking groove of SRPK2 modulates Cp binding and phosphorylation

**Fig 2:**
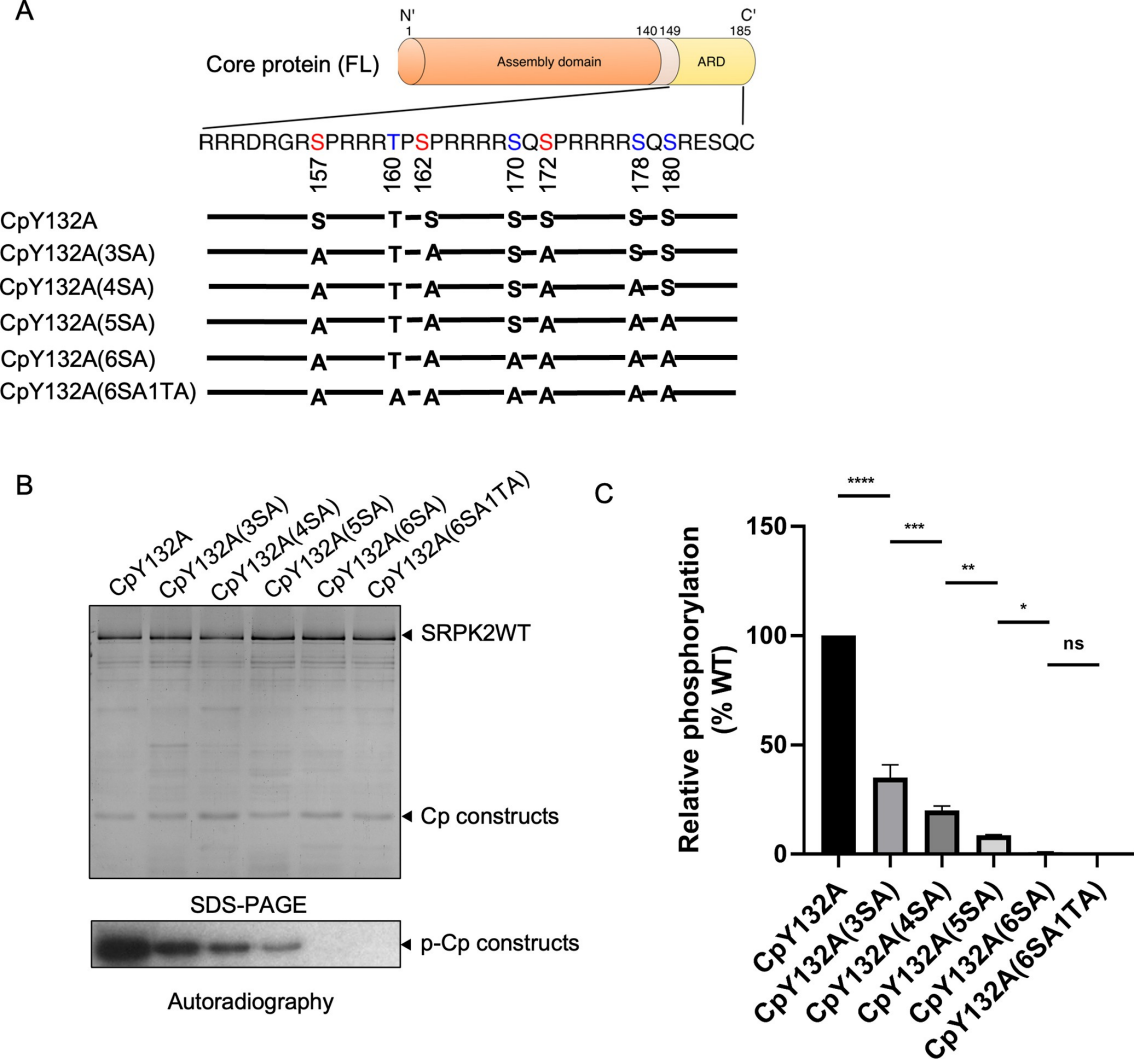
SRPK2 phosphorylation sites in the ARD of Cp. (A) Domain organization of Cp. The potential phosphorylation sites in the ARD are indicated. The major phospho-acceptors (S157, S164, and S172) are colored in red whereas the minor phospho-acceptors (T162, S170, S178, and S180) are colored in blue. The mutational constructs generated for the *in vitro* kinase assays are shown. (B) Cp contains six SRPK2-mediated phosphorylation sites. *In vitro* radioactive kinase assay was performed using SRPK2WT and the mutational Cp constructs shown in (A) in the presence of [^32^P]ATP. Reactions were quenched after 10 mins and resolved by SDS-PAGE. The gel was visualized with Coomassie Blue staining and then subjected to autoradiography. Mutation of six serines to alanines abolished the phosphorylation of Cp by SRPK2 (C) Phosphorylation levels of different Cp mutants relative to wild type Cp. Radiolabeled protein bands corresponding to the phosphorylated Cp mutants were excised and quantified by scintillation counting. Data are expressed as mean ± S.D. for three independent experiments. One-way ANOVA test was used for statistical analysis. *, p < 0.05; **, p < 0.005; ***, p < 0.001; ****, p < 0.0001.

Although SRPK2 is suggested to play a regulatory role during HBV replication [30], molecular mechanisms underlying the interaction and phosphorylation of Cp by SRPK2 remain unclear. We identified possible SRPK2 phosphorylation sites within the ARD of Cp to determine whether SRPK2 phosphorylates the same sites as SRPK1 [31]. On the basis of the seven conserved phosphorylation sites identified within the ARD previously [21], a CpY132A mutant containing alanine substitutions at S157, S164, and S172, which are considered the major phosphorylation sites, was constructed. Four other mutants, each containing an additional alanine substitution at the other identified phosphorylation sites, were also generated (Fig 2A). *In vitro* kinase assays revealed that SRPK2 phosphorylated six sites in the ARD of the CpY132A but did not phosphorylate T160 (Fig 2B), which was determined as an SRPK1 phosphorylation site previously. Mutation of the residues S157, S164, and S172 markedly reduced the phosphorylation content by approximately 60% of that of the wild type (Fig 2C); this finding is in accordance with those of previous studies.

Next, we investigated how SRPK2 recognizes and interacts with Cp. SRPK2 is a close homolog to SRPK1, sharing over 90% sequence similarity in their kinase core domains. However, their nonkinase regions are not homologous. To determine whether the non-homologous N-terminal and spacer regions play a role in the binding of SRPK2 to Cp, we truncated these regions independently or together and performed a GST pull-down assay by using GST–CpY132A as the bait (S1 Fig). Our results revealed that all truncation constructs could still bind to the CpY132A, suggesting that the nonkinase regions of SRPK2 are not essential for the interaction.

**Fig 3:**
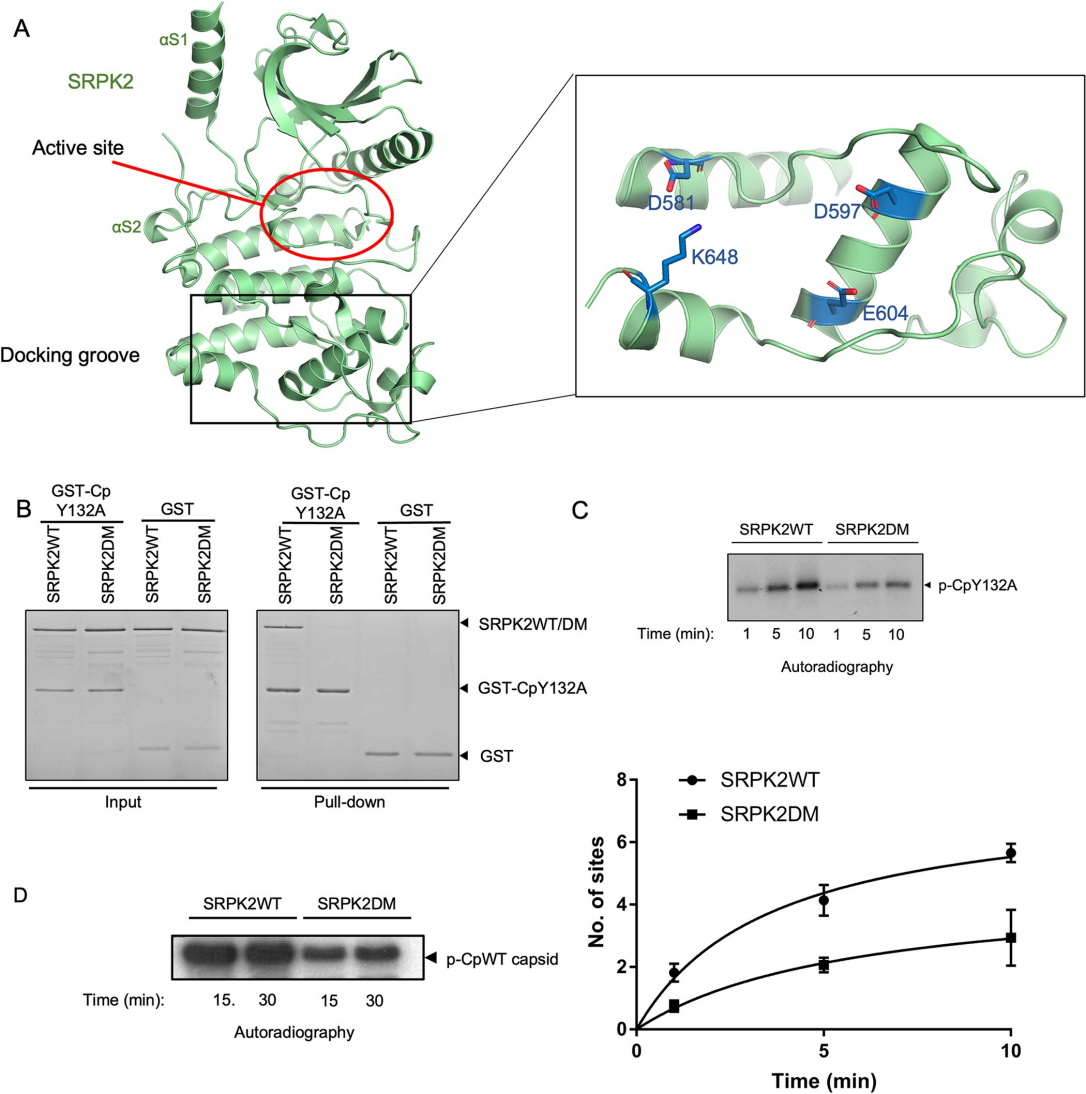
Docking groove of SRPK2 governs the binding and phosphorylation of Cp. (A) Crystal structure of SRPK2ΔNS (PDB ID: 5MYV). A SRPK-specific docking groove is located at the large lobe of the kinase. Active site of the kinase is indicated. Four amino acid residues that are critical for substrate binding and phosphorylation are indicated (right). (B) *In vitro* GST pull-down assay of GST-tagged CpY132A with SRPK2WT or SRPK2DM. Free GST was used as a control. Samples were resolved by SDS-PAGE and stained by Coomassie Blue. Mutations at the docking groove of SRPK2 abolished the binding of Cp. (C) *In vitro* radioactive kinase assays with SRPK2WT or SRPK2DM and CpY132A were performed for the indicated time points in the presence of [^32^P]ATP. Samples were resolved by SDS-PAGE and visualized by autoradiography (upper panels). Radiolabeled protein bands corresponding to phosphorylated CpY132A were excised and quantified by scintillation counting. Data are expressed as mean ± S.D. for three independent experiments (lower panel). (D) *In vitro* radioactive kinase assays with SRPK2WT or SRPK2DM and CpWT capsid for extended periods of time. The reactions were analyzed by SDS-PAGE and visualized by autoradiography. SRPK2DM failed to phosphorylate CpWT capsid to the same extent as SRPK2WT.

SRPKs contain a conserved electronegative docking groove in kinase domain that is distal to the catalytic site. Four conserved amino acids within the docking groove, namely D548, D564, E571, and K615 in SRPK1 and D581, D597, E604, and K648 in SRPK2, are pivotal for the binding and phosphorylation of different SR-rich substrates [25–28] (Fig 3A). To examine whether the docking groove residues of SRPK2 are crucial for Cp binding, we mutated the four key residues to alanine (SRPK2DM) and investigated their effect on Cp binding by performing a GST pull-down assay. We observed that the docking groove mutations abolished the binding of the GST–CpY132A and attenuated its phosphorylation (Fig 3B and 3C). Furthermore, the SRPK2DM mutant was less effective in the phosphorylation of CpWT in its capsid form even after we prolonged the phosphorylation reaction to 30 mins (Fig 3D), suggesting that the docking groove–mediated interaction of Cp governs its phosphorylation mechanism. Although SRPK2DM failed to bind CpY132A stably, the mutant protein might still interact with Cp weakly and transiently to phosphorylate it in an unregulated manner. These results demonstrated that apart from canonical SR-rich substrates, the binding and phosphorylation of Cp depend on the docking groove of SRPK2.

### Mechanism underlying the phosphorylation of Cp by SRPK2

The conserved docking grooves of SRPKs are vital for the regulation of the phosphorylation mechanisms of different substrates. In general, a basic residue-rich region N-terminal to phosphorylatable RS dipeptides functions as a docking motif to interact with the docking groove of SRPK and precisely positions the first phosphor-accepting residue at the kinase active site to initiate phosphorylation. The ARD of Cp contains multiple basic regions that precede the phosphorylation sites, but not all its phosphorylatable residues are located adjacent to an arginine. Thus, we speculate that the phosphorylation mechanism of Cp is unique from that of other SR-containing substrates. We first identified the regions within the ARD of Cp that can serve as a docking motif to interact with SRPK2 (S2A Fig). We generated four CpY132A mutants, each with stretches of arginine N-terminal to a phosphorylation site mutated to alanine (S2B Fig). GST pull-down assay revealed that the arginine-rich region 150–156 (M1) was crucial for SRPK2 binding, indicating that this region might serve as a docking motif during substrate recognition (S2C Fig). However, the mutations of neither this region nor other arginine-rich regions significantly affect the phosphorylation of the core protein (S2D Fig).

**Fig 4:**
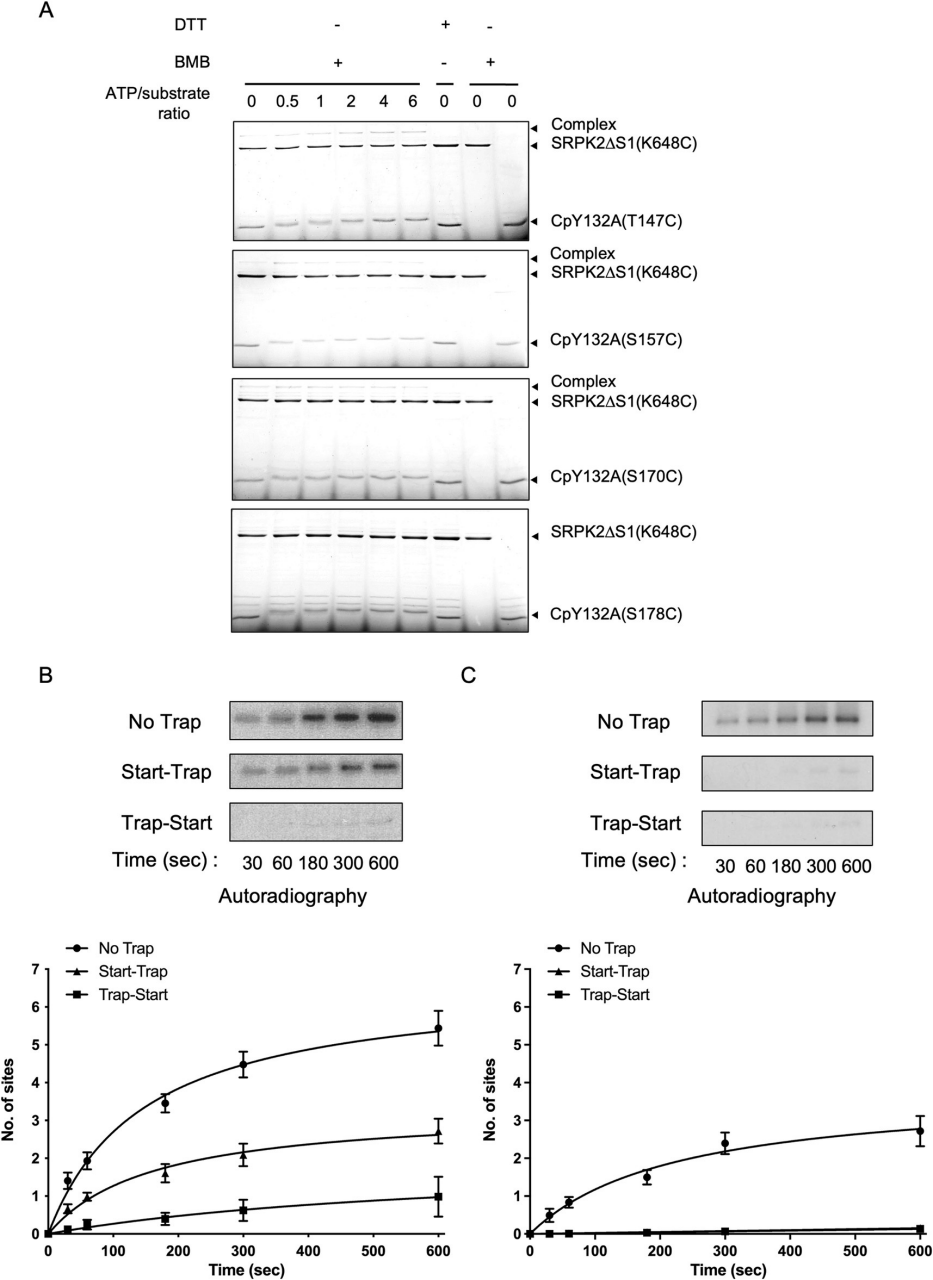
Mechanism underlying Cp phosphorylation by SRPK2. (A) Disulfide crosslinking between SRPK2 and Cp. SRPK2ΔS1(K648C), which contains a cysteine at the docking groove, and CpY132A mutants containing cysteine mutations at different positions within the ARD were allowed to crosslink by BMB for 15 mins. Reactions were performed in the presence of increasing concentrations of ATP to observe how different extent of phosphorylation affected the crosslinking. The molar ratio of ATP to Cp is indicated. The reactions were analyzed by SDS-PAGE with Coomassie staining. CpY132A(T147C) crosslinked with SRPK2 in an ATP-dependent manner whereas CpY132A(S157C) and CpY132A(S170C) crosslinked with SRPK2 regardless ATP was present or not. CpY132A(S178C) failed to crosslink. (B, C) Phosphorylation of CpY132A by (B) SRPK2WT and (C) SRPK2DM under different trapping conditions. In the start-trap reaction, SRPK2WT or SRPK2DM was pre-incubated with CpY132A and then allowed to react with [^32^P] ATP in the presence of excess trap (SRPK2ΔS1(K110M), a kinase-dead mutant). No trap reaction, which served as a positive control, was performed in the same manner in the absence of SRPK2ΔS1(K110M). Trap-start reaction, which served as a negative control, was performed by preincubating SRPK2ΔS1(K110M) and CpY132A before the addition of SRPK2WT or SRPK2DM. Reactions were quenched at different time points. Samples were resolved by SDS-PAGE followed by autoradiography. Radiolabeled protein bands corresponding to phosphorylated CpY132A were excised and quantified by scintillation counting. Data are expressed as mean ± S.D. for three independent experiments.

We investigated whether the observed result can be attributed to a sliding mechanism as determined for other substrates of SRPKs, where different docking motifs present within a substrate can bind to the docking groove to compensate for the loss of the initial binding site and enable phosphorylation to continue. We performed mutation-directed disulfide crosslinking experiments to test the hypothesis (Fig 4). In brief, all solvent-exposed cysteines on SRPK2ΔS1, an active SRPK2 construct in which the nonessential spacer domain was truncated, and the CpY132A were mutated to serines to prevent nonspecific crosslinking. Four strategic sites located at different sections of the ARD, namely T147, S157, S170, and S178, were mutated to cysteine in the context of the surface cysteine-free CpY132A independently. In addition, K648 of SRPK2, which corresponds to K615 of SRPK1 that interacts with the docking motif’s backbone but not the alternating arginines, was mutated to cysteine in the surface cysteine-free kinase due to its close proximity to the docking motif (S3A and S3B Fig). The kinase and CpY132A mutants were then allowed to crosslink in the presence of the crosslinker 1,4-bismaleimidobutane (BMB) and different ATP concentrations, which were added to control the sliding of the substrate, if any, during the multisite phosphorylation reaction. A control experiment using SRPK2ΔS1(K648C) and CpY132A(surface C to S) were performed to validate the specificity of the crosslinking reaction (S3C Fig). Western blots were also performed to confirm the formation of complexes upon the addition of BMB and ATP (S4 Fig). Both CpY132A(S157C) and CpY132A(S170C) crosslinked with SRPK2 in an ATP-independent manner (Fig 4A). Although CpY132A(T147C) also weakly crosslinked with SRPK2, more crosslinked products were observed when the ATP concentration was increased. By contrast, CpY132A (S178C) failed to crosslink with SRPK2, regardless of the addition to ATP (Fig 4A). These results indicate that although SRPK2 did not strictly bind to a single docking motif on Cp, it preferentially interacted with the middle region of the ARD prior to phosphorylation. On the other hand, T147 at the N-terminal region of the ARD gained more access to the docking groove only after the initiation of the phosphorylation event, suggesting that the ARD likely moved along the docking groove in a C-to-N manner during phosphorylation.

Because the substrate could bind to the docking groove in a mobile manner, we performed start-trap assays to investigate whether CpY132A is phosphorylated by SRPK2 in a processive manner. In brief, excess kinase-dead SRPK2 mutants (trap) were added to the reaction after the initiation of CpY132A phosphorylation (start). If the phosphorylation reaction was processive, the presence of excess traps would not affect the phosphoryl content of the substrate. Our results revealed that SRPK2 phosphorylated the CpY132A at approximately six sites in the absence of trap. By contrast, 2.7 ± 0.3 sites were phosphorylated under the start-trap condition (Fig 4B), suggesting that CpY132A was phosphorylated in a processive manner, in which the substrate molecules remained associated with the kinase after nearly three phosphorylation events. Next, we examined whether the kinase docking groove is crucial for the processive phosphorylation. The SRPK2DM mutant failed to phosphorylate the CpY132A in the presence of trap (Fig 4C), underscoring the crucial role of the docking groove in Cp phosphorylation.

### SRPK2 exhibits a kinase-gated mechanism to prevent core assembly

**Fig 5:**
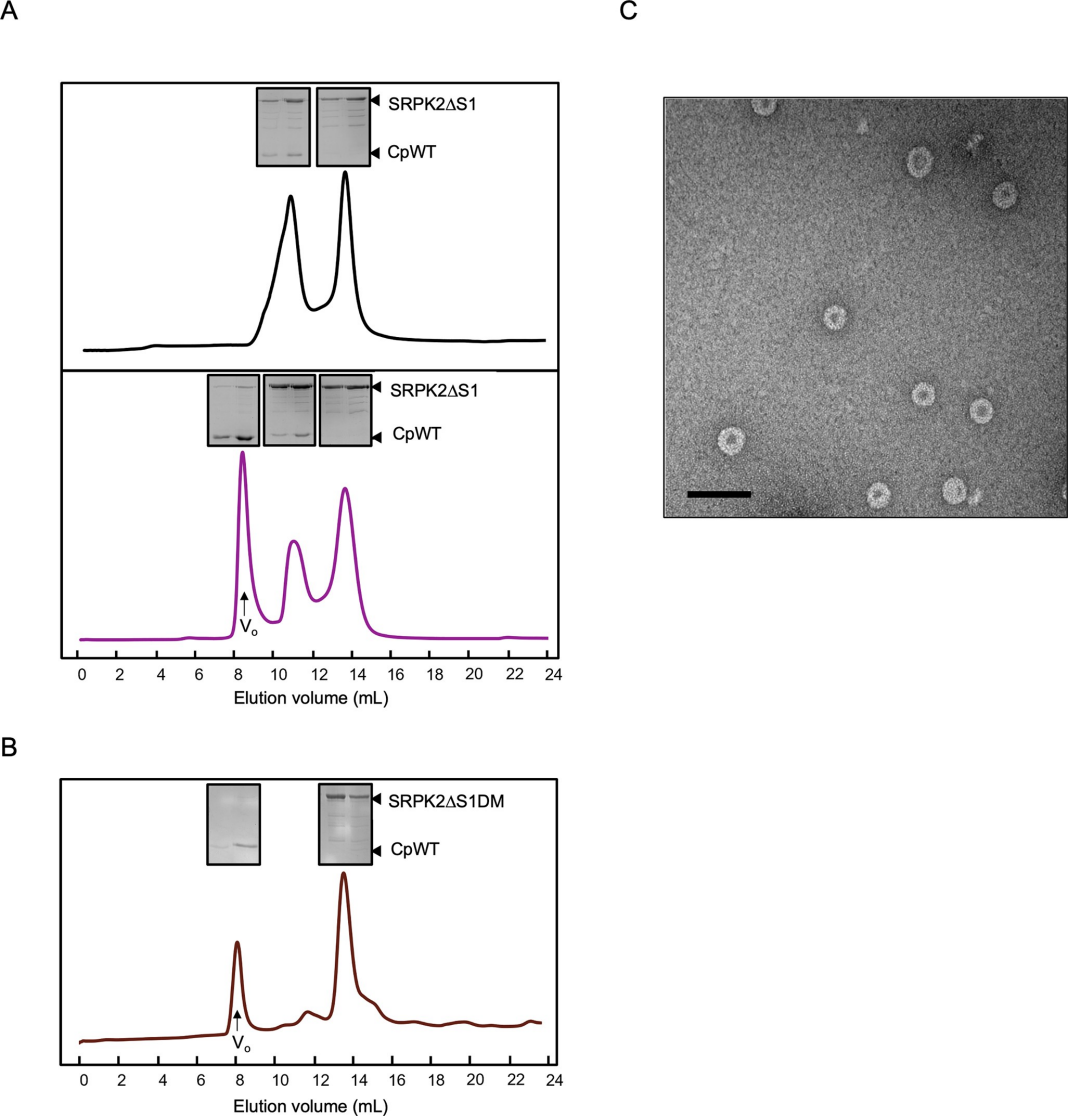
Size-exclusion chromatography analysis of SRPK2-CpWT complex. **(A)** Size-exclusion chromatograph of CpWT after the removal of GuHCl by dialysis. All CpWT assembled into capsids and eluted at the void volume (V_o_) (B) SRPK2 prevented capsid self-assembly. Size-exclusion chromatographs show that SRPK2ΔS1 and CpWT dimer formed a stable complex and was eluted at ~11 mL after removal of GuHCl; excess kinases were eluted at ~13 mL (black, upper panel). Addition of ATP/Mg^2+^ resulted in capsid assembly, indicated by an additional peak at V_o_ (purple, lower panel). The insets show the SDS-PAGE analysis of the peak fractions. Capsid assembly was induced upon SRPK2-mediated phosphorylation. (C) Negative staining of the peak fraction collected at ~8.5 mL of the reaction with ATP/Mg^2+^ (lower panel in (A)). The diameter of the capsid is about 35 nm. The scale bar is 100 nm. (D) Docking groove of SRPK2 is crucial for regulating capsid self-assembly. Size-exclusion chromatograph (brown) shows that no complex was formed after removal of GuHCl. Reassembled capsid was eluted at void volume (V_o_) while excess SRPK2ΔS1DM was eluted later. The mutations at the docking groove dampened the assembly of capsids.

Cp self-assembled into capsids independent of its phosphorylation or the presence of the viral pregenome [15,32]. We have performed size-exclusion chromatography using our purified wild-type core protein (CpWT) and showed that it also self-assembled into capsids in the absence of GuHCl (Fig 5A). A recent study proposed that SRPK1 gates the core assembly process by binding to unphosphorylated Cp to prevent premature self-assembly. Cp is released to encapsidate the viral pregenome only upon phosphorylation. To investigate whether SRPK2 exhibits a similar mechanism to regulate HBV capsid formation, we analyzed the formation of the SRPK2–Cp complex by performing size-exclusion chromatography using SRPK2ΔS1 and CpWT in the presence or absence of ATP as described by Zlotnick and colleague [15]. In brief, excess SRPK2ΔS1 was first incubated with CpWT together with a low concentration of guanidine hydrochloride (GuHCl) to maintain the latter in a dimeric form and prevent premature capsid assembly. GuHCl was slowly dialyzed away, and complexes formed before and after the addition of ATP and Mg^2+^ were subjected to size-exclusion chromatography. Our results revealed that in the absence of ATP, all core proteins were trapped by SRPK2 to form heterodimeric complexes (Fig 5B, upper panel). However, in the presence of ATP, although a portion of core proteins remained bound with a small amount of SRPK2ΔS1, they were eluted in the void volume (Fig 5B, lower panel), suggesting that they had assembled into high-molecular-weight capsids. To confirm the formation of capsid after addition of ATP, the void volume was collected and subjected to negative EM imaging. It revealed that the samples had assembled into spherical particles with a diameter of approximately 35 nm, which is the typical diameter of a T = 4 HBV capsid, confirming the formation of capsid after the addition of ATP/Mg^2+^ (Fig 5C). This finding suggested that SRPK2, similar to SRPK1, might gate the core assembly by binding to unphosphorylated Cp and maintaining it in a low oligomeric state. Subsequently, the phosphorylation of Cp by SRPK2 triggered the dissociation of most of the kinases and allowed the formation of HBV capsids. Next, we analyzed the role of the docking groove in SRPK2 in this gating mechanism. Size-exclusion chromatography analysis results indicated that the mutant failed to form a stable complex with CpWT dimer and did not prevent capsid formation even when the ATP was absent, indicating the importance of the docking groove in regulating capsid assembly (Fig 5D).

### SRPK2 binds to the same sites on the HBV capsid as SRPK1

**Fig 6:**
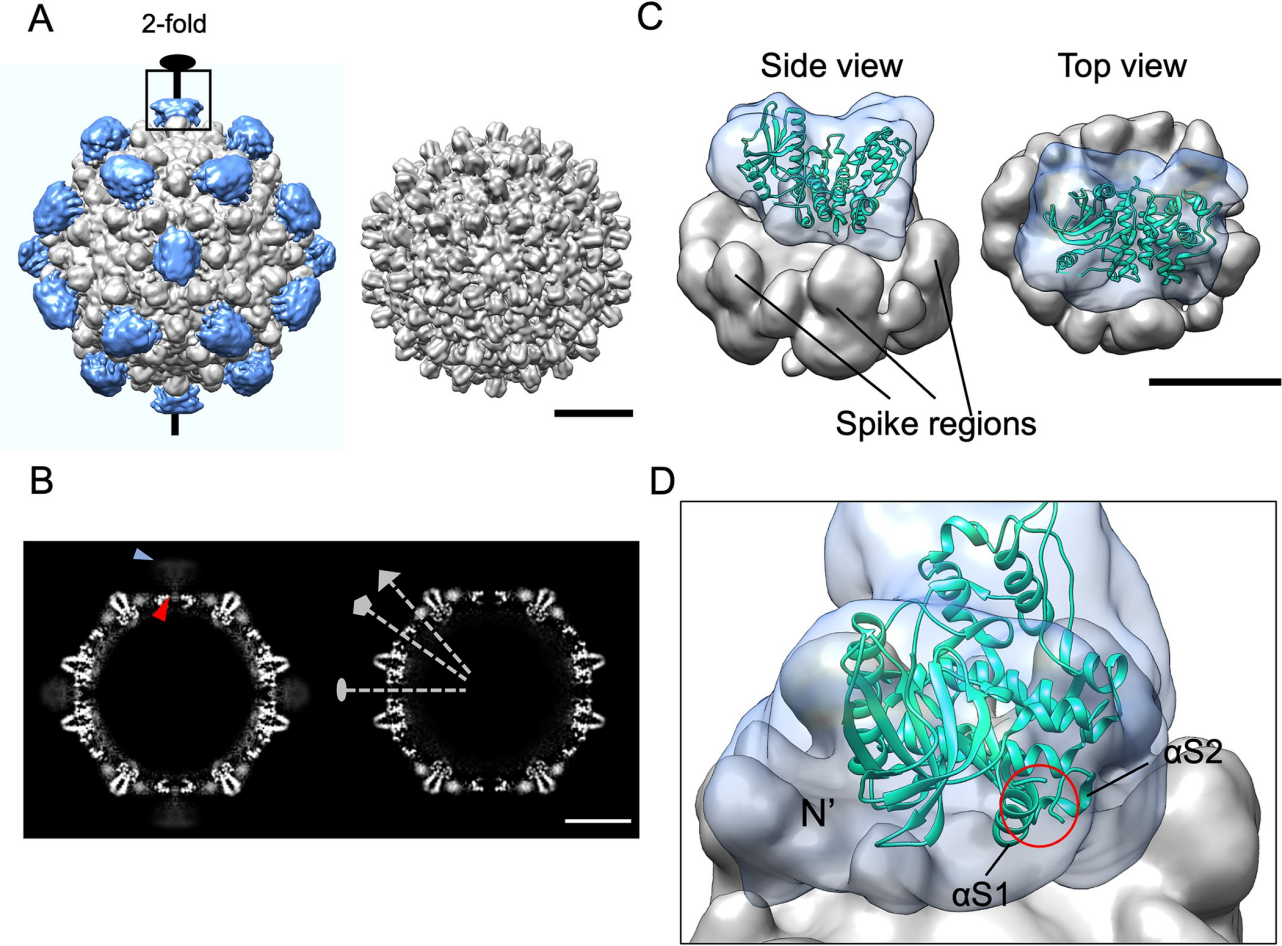
Reconstructions of CpWT capsid and the complex with SRPK2ΔS1. (A) Surface representations of the reconstructed SRPK2ΔS1/CpWT capsid complex (left) and apo-capsid (right). Kinases that decorated the 2-fold vertices of the capsid are colored in blue. Scale bar represents 10 nm. All views are along the icosahedral twofold axis. Both maps are low-pass filtered to 10 Å. (B) Central slice of the complex (left) shows a protruding density at the 2-fold vertex (indicated by blue arrowhead) and a stretch of density that extend to the presumably surface-bound kinase via the pore (indicated by the red arrowhead). The 2-fold, 3-fold and 5-fold symmetry axes are indicated by the grey dashed lines with oval, triangular and pentagonal symbols respectively in the central slice of apo-capsid (right). Scale bar represents 10 nm. (C) Segmentation and model fitting of the focus-refined SRPK2ΔS1/CpWT capsid at the 2-fold vertex. The capsid and SRPK2ΔS1 densities are colored in light grey and blue, respectively. Crystal structure of SRPK2 (PDB ID: 5MYV) was used in model fitting. Scale bar represents 5 nm. (D) Model fitting showed some densities of the reconstruction near the missing regions (a.a. 236–256, 507–511) between the spacer helices αS1 and αS2 of SRPK2ΔNS in the crystal structure. The missing regions are indicated by the red circle; extra densities are observed near the N-terminal region of the kinase.

Because our size-exclusion chromatography results revealed that a small amount of SRPK2 remained bound to the Cp after capsid assembly (Fig 5B), we performed immunogold analysis on the eluted SRPK2ΔS1/CpWT capsid particles. Our result confirmed that SRPK2 were presented on the capsid particles (S5 Fig). Therefore, we solved the cryo-EM structure of the HBV capsid in complex with SRPK2. Both SRPK2ΔS1 and CpWT dimer were expressed with N-terminal His-tag and purified using Ni-NTA affinity chromatography; complexes of SRPK2ΔS1 bound to CpWT dimer or CpWT capsid were formed and further purified by gel filtration (S6 Fig). The reconstruction of the HBV capsid and SRPK2ΔS1/CpWT capsid complex applied with icosahedral symmetry resulted in maps with overall resolutions at 4.4 Å and 4.6 Å respectively, and the quality of the maps was validated by the fitting of the atomic model of CpWT (S7 Fig). We compared the cryo-EM structures of the CpWT capsid in its apo form and in complex with SRPK2ΔS1. The 2D class averages of SRPK2ΔS1/CpWT capsid complex showed extra surface density when compared to the apo form (S8 Fig). We reasoned that the extra density belonged to the SRPK2ΔS1 protein. Weak and fuzzy density of SRPK2ΔS1 suggested structural flexibility and low occupancy of SRPK2ΔS1 on capsid. A similar observation was reported for the cryo-EM structure of the SRPK1–capsid complex [15]. A comparison of the segmented 3D reconstructions of the complex and apo-capsid after low-pass filtering to 10 Å revealed SRPK2ΔS1 densities (colored in blue) at the 2-fold vertices of the complex reconstruction (Fig 6A). In addition, we observed that some densities (indicated with a red arrowhead) extend through the 2-fold vertex to the capsid surface where SRPK2ΔS1 was located (indicated with a blue arrowhead) (Fig 6B, left). We attribute these densities to the SRPK2ΔS1-bound externalized CTD of CpWT capsid, which can be exposed to the capsid surface transiently. The central slice of the apo-capsid reconstruction revealed that the opening at the icosahedral 2-fold vertex was larger than the one at the 5-fold vertex (Fig 6B, right). The extra density observed at the 3-fold vertices was the N-terminal polyhistidine tag of our CpWT construct since the N-terminal ends of core proteins localize at the same site [19] (S7C Fig).

To further elucidate the structural information about the SRPK2 on capsid, we performed focused classification to mitigate structural heterogeneity. In brief, particles from the previous reconstruction were expanded with I symmetry, followed by particle subtraction retaining signal from SRPK2ΔS1/CpWT capsid complex at one 2-fold vertex with a reference mask covering the whole kinase density and some surrounding densities in the capsid region, and 3D classification with regularization parameter T of 40 and local angular search (S9 Fig). A class of 58,259 sub-particles was chosen for the subsequent masked 3D auto-refinement with the applied C1 symmetry. The 3D density map of SRPK2ΔS1/CpWT complex at 2-fold vertex was reconstructed at a resolution of 11 Å (S7D Fig). Segmentation of the focus-refined density map was performed, and densities representing the 2-fold vertex of the CpWT capsid and SRPK2ΔS1 are colored in light gray and blue, respectively. The crystal structure of the kinase domain of SRPK2 was fitted into the focus-refined density map using Chimera (Fig 6C). The model revealed that the large lobe of SRPK2 lies close to the opening at the 2-fold vertex. Some extra densities of the reconstruction were located near the spacer helices αS1 and αS2 of SRPK2 (Fig 6D); this finding was likely due to parts of the spacer region of SRPK2 that were retained in our construct but was disordered in the crystal structure model. Another region of extra densities could be ascribed to the N-terminal region of our SRPK2 construct (Fig 6D), which was not observed in the crystal structure model. Although the externalized CTDs of Cp may extend into the density maps of kinases, we could not solve the structure of the binding interface between the two proteins due to low resolution. However, our size-exclusion chromatography result for SRPK2DM and CpWT capsid suggested that the binding of SRPK2 on the capsid surface is dependent on the direct interaction between the kinase docking groove and the ARD of Cp.

Our results demonstrated that the two members of the SRPK family can bind to the 2-fold vertices of the HBV capsid. We examined whether the ARD of CpWT capsid remains phosphorylable by SRPKs after capsid formation and how the simultaneous presence of both SRPK1 and SRPK2 affects the phosphorylation event. We performed an *in vitro* kinase assay to examine the phosphorylation of CpWT capsids by SRPK1, SRPK2, or both (S10 Fig). Excess kinases were used to ensure single-turnover conditions. Our results revealed that SRPK2 phosphorylated CpWT capsids more effectively than did SRPK1. Moreover, when both kinases were added to the reaction, the phosphoryl content of CpWT capsids was lower than that observed when only SRPK1 or SRPK2 was added. This finding indicates that SRPK2 and SRPK1 might compete for the same phosphorylation sites on the capsid surface, supporting the observation that both kinases bind to the same vertices of the HBV capsid.

### SRPK2 is important for Cp phosphorylation in cells

**Fig 7:**
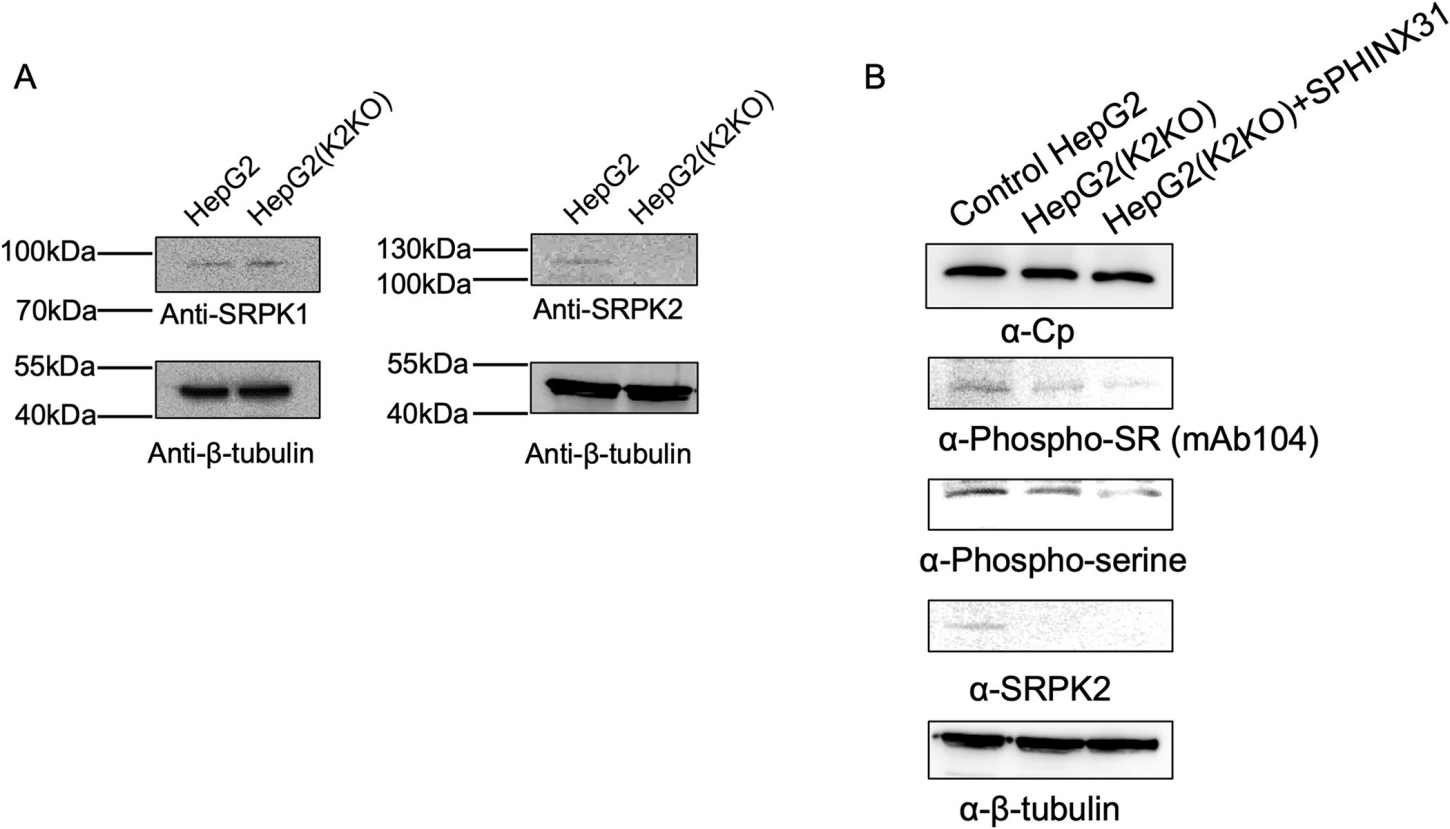
SRPK1 and 2 are important for the phosphorylation of Cp. (A) Generation of SRPK2-knockout HepG2 cell line. Western blot showed the expression level of SRPK1 was similar in both control HepG2 and HepG2(K2KO) cell lines whereas SRPK2 was absent in HepG2(K2KO) cells. (B) Knockout of SRPK2 and further inhibition of SRPK1 suppressed the phosphorylation of Cp. Both control HepG2 and HepG2(K2KO) cell lines were transfected with pcDNA3.1-myc-His-Cp. Transfected cell lysates were incubated with Ni-NTA resins to pull down His-Cp. Western blots showed that the phosphorylation level of Cp was reduced upon SRPK2 knockout. Inhibition of SRPK1 in HepG2(K2KO) cells with SPHINX31 further inhibited the phosphorylation of Cp.

To confirm whether SRPK2 is truly a cellular kinase that phosphorylates Cp, we constructed a stable SRPK2-knockout HepG2 cell line (HepG2(K2KO)) by using the CRISPR-Cas9 gene-editing system. Knockout of SRPK2 was confirmed through Western blotting and DNA sequencing (Figs 7A and S11). Levels of endogenous SRPK1 in the control and knockout HepG2 cells were comparable, indicating that the absence of SRPK2 did not alter SRPK1 expression. We first performed *in vitro* phosphorylation assay using recombinant GST-CpWT and cell lysates from control HepG2 and HepG2(K2KO) cells to test if endogenous SRPK2 is important for Cp phosphorylation (S12 Fig). Our result demonstrated that the knockout of SRPK2 reduced the phosphorylation of GST-CpWT, suggesting cellular SRPK2 is important for the phosphorylation event.

Next, we transfected both control HepG2 and HepG2(K2KO) cells with CpY132A to investigate the effects of SRPK2-knockout on Cp phosphorylation in cells. Knockout of SRPK2 reduced the Cp phosphorylation level (Fig 7B). When HepG2(K2KO) cells were treated with the SRPK1-selective inhibitor SPHINX31 to inhibit SRPK1 [33], phosphorylation of Cp was further reduced. These results provided further evidence that SRPKs are important cellular kinases for Cp phosphorylation.

## Discussion

Several cellular kinases, including cdc2 kinase, PKC, PKA, a 46-kDa serine kinase, and SRPKs, have been implicated in the phosphorylation of Cp [21,34–37]. Among these kinases, SRPKs are considered as the major kinases, and SRPK2 might play a more crucial role given its higher binding affinity and activity toward Cp [21]. The present study provided further evidence that SRPK2 plays important role in the phosphorylation of Cp and potentially regulates capsid assembly; and demonstrated the molecular mechanism through which SRPK2 binds and phosphorylates Cp. Although our *in vitro* biochemical analysis indicated that SRPK1 and SRPK2 bind to Cp with comparable affinities, our results indicated that SRPK2 exhibited higher kinase activity toward Cp. This finding suggests that the previously observed stronger binding between SRPK2 and Cp could be due to an additional regulatory mechanism. For instance, SRPKs can shuttle between the cytoplasm and nucleus, and their nuclear import is regulated by different signal transduction pathways and posttranslational modifications [38–41]. The differential subcellular localization of the two kinases might affect their interactions with Cp in a temporal and spatial manner during different stages of the viral life cycle.

Cp is a noncanonical substrate of SRPKs because its ARD contains only four RS/SR dipeptides and three SPRRR motif repeats. Serines within the three SPRRR motifs (S157, S164, and S172) are major phosphor-acceptors [20, 42], whereas the other four residues are considered as minor phosphorylation sites (T162, S170, S178, and S180) [22,36,43,44]. Among these, only S157, S170, S178, and S180 are related to RS/SR dipeptides. Consistent with the findings of previous studies, our results demonstrated that SRPK2 mainly phosphorylated S157, S164, and S172, where both S164 and S172 are not adjacent to an arginine. This finding suggests that the presence of an arginine at the P+1 site is not essential for SRPKs. In contrast to the previous finding that SRPK1 phosphorylates seven residues, including T162 within the CTD of Cp, when the two proteins are co-expressed in *E*. *coli* [31], our kinase assays indicated that the alanine substitutions of the six serines abolished phosphorylation by both SRPK2 and SRPK1 *in vitro* (S12 Fig). Although T162 is phosphorylatable by SRPK1 when Cp is exposed to the kinase for a prolonged period, it might not be a major phosphorylation site for SRPKs because of their preference for serine over threonine residues. Furthermore, the nonhomologous kinase regions that were truncated in the co-expression study might play a role in the different specificities observed in this study. For instance, the N terminus of SRPK2 is crucial for the phosphorylation of the SR-like protein Acinus both *in vitro* and *in vivo* [27]. Because the phosphorylation of T162 is crucial for the synthesis of the minus strand, plus strand, and rcDNA [22], other cellular kinases are likely required to coordinate with SRPKs to regulate the dynamic phosphorylation of Cp.

Our previous studies have demonstrated that a conserved substrate-docking groove in SRPK governs the binding and phosphorylation mechanisms of different SR-containing substrates [25–28]. In this study, we determined that such docking groove plays a critical role in the binding and phosphorylation of Cp. Similar to other classical SR proteins such as SRSF1 and SRSF3, SRPK2 phosphorylates Cp at nearly half of the phosphorylation sites in a processive manner given the presence of multiple docking motifs within the ARD [26]. These docking motifs enable the initiation of Cp phosphorylation at different sites through a mobile docking mechanism in which the mid-region of the ARD first binds to the kinase and then moves in a C-to-N direction. Through this mechanism, SRPK2 not only may regulate the electrostatic potential of Cp by phosphorylating multiple sites but also may secure its CTD at the docking groove and block it from interacting with the pregenome or other nonspecific RNA, thus preventing premature encapsidation before the completion of ARD phosphorylation.

Like the C-to-N movement of the CTD of Cp, the RS domains of both SRSF1 and SRSF3 are also phosphorylated by SRPK2 in a C-to-N manner [26]. However, contrary to the tandem arrangement of RS dipeptides in the classical SR proteins, the phosphorylation sites in Cp are discontinuous. Our results prove that the docking groove of SRPK2 is capable of regulating diverse phosphorylation mechanisms of different substrate classes. Additional structural studies should be conducted to determine how the docking groove of SRPK2 mediates the interaction with its very different substrates.

HBV nucleocapsid formation involves capsid assembly and viral pregenome encapsidation [45,46]. During capsid assembly, the core protein dimer undergoes nucleation, followed by the aggregation and arrangement of 120 copies into a T = 4 icosahedral capsid [47]. Capsid assembly is a spontaneous process that occurs even in the absence of the viral pregenome, and Cp can still assemble into an empty capsid. Zlotnick and colleagues [15] demonstrated that SRPK1 regulates nucleocapsid formation through a gating mechanism, where the kinase binds to unphosphorylated Cp to prevent their self-assembly. Upon phosphorylation, SRPK1 releases Cp for capsid formation. Our current study indicated that SRPK2 adopts the same kinase-gated mechanism to regulate Cp assembly. Moreover, such mechanism is dependent on both the docking and phosphorylation of Cp by SRPK2, implying a new functional role of the conserved docking groove in viral infection. A previous study reported that the overexpression of SRPKs and their inactive counterparts in HBV-infected cells suppresses pgRNA encapsidation through a phosphorylation-independent pathway [30]. Such findings can be explained by our observation that both SRPKs can remain bound to Cp through docking interaction, albeit with different affinities, regardless of their phosphorylation state.

Our cryo-EM studies revealed that similar to SRPK1, SRPK2 binds to the surface of the assembled capsid particle. Because the interaction between SRPK2 and Cp is dependent on the ARD of Cp binding to the kinase docking groove, the inner CTD tails of Cp can externalize and become exposed to the surface environment [48]. Both SRPK2 and SRPK1 are anchored to the same 2-fold vertices of the capsid particle, suggesting that the two kinases may compete for the same binding sites; this speculation is supported by the results of our *in vitro* kinase assay. The Cp CTD tails cluster at both 2- and 5-fold vertices [19]. The reason for the nonbinding of SRPKs to CTD tails at the 5-fold vertices remains unclear. However, the efficiency of CTD externalization might be limited by the smaller pore size at the 5-fold vertices [49]. In our model, we observed the docking groove of SRPK2 faces the side opposite the capsid. Nonetheless, empty space between the densities of SRPK2 and the capsid may allow the flexible externalized Cp CTD to wrap around the kinase large lobe to access the docking groove. During the phosphorylation event, SRPK2 is speculated to undergo orientational and conformational changes to allow the sliding of the ARD of Cp along the kinase docking groove to facilitate multi-site phosphorylation. Such mechanism might be vital for viral replication because interactions among host factors, viral genetic materials, and Cp are dependent on the CTD phosphorylation state and require dynamic phosphorylation and dephosphorylation during nucleocapsid formation [20,31,43,50]. In addition, rephosphorylation of core particles before nuclear import (or re-import) is observed [51]. Given that both SRPK1 and SRPK2 are unlikely to play an equivalent role in Cp phosphorylation, future studies should investigate when and which SRPKs bind to Cp during the different stages of the viral life cycle.

Our cell-based results revealed that the knockout of SRPK2 and inhibition of SRPK1 reduced the phosphorylation of Cp, implying the role of SRPK2 in the HBV life cycle. However, further studies using infectious models are needed to validate the roles of SRPK1 and 2 in HBV replication and the pharmaceutical value of the docking groove of SRPK as a target in combating HBV replication and infection.

## Materials and methods

### Plasmid constructions

DNA inserts containing sequence of full-length HBV core protein (subtype: adw2) were cloned into pGEX-4T-2 and pET-15b. Dimer form of core protein is made based on the Y132A mutation as reported previously [29]. Core protein constructs with different combinations of Ser/Thr to Ala mutation were derived from the plasmid encoding N-terminally His-tagged full-length core protein in Y132A background using site-directed mutagenesis. N-terminally GST-tagged core protein constructs with different arginine-rich motifs mutated to alanine were purchased and synthesized from GenScript. Full-length SRPK2 were cloned into pET-15b for the purification of SRPK2 with N-terminal His-tag. Different truncation constructs of SRPK2 were derived from the plasmid encoding full-length SRPK2. Expression plasmids pcDNA-myc-His-Cp was made by cloning the sequence of CpY132A with into pcDNA3.1(+) myc/His A vector.

### Expression and purification of recombinant proteins

Different GST-Cp constructs were overexpressed in *E*. *coli* strain, BL21(DE3)pLysS. Transformed bacterial was grown in LB broth at 37°C and then induced with 0.2 mM IPTG at 16°C for 18 hours when O.D._600_ = 0.6. Cells were collected and lysed in lysis buffer (20 mM Tris,150 mM NaCl, 10 mM DTT, 5% glycerol, pH 7.4) by sonication. Cell lysate was centrifuged at 20,000 x g for 1 hour. Supernatant was then applied to GST-chromatography in which glutathione resin (GenScript) had been equilibrated in the lysis buffer. After extensive wash with the lysis buffer, Bound proteins were eluted by elution buffer (20 mM Tris, pH 7.4, 150 mM NaCl, 10 mM DTT, 5% glycerol, 60 mM reduced glutathione). Different fractions (flow-through, wash, elution) were collected and analyzed by SDS-PAGE. Gel was stained by Coomassie Blue. Eluted proteins were dialyzed against dialysis buffer (20 mM Tris, pH 7.4, 150 mM NaCl, 10 mM DTT, 5% glycerol) and concentrated and frozen at -80°C. Different constructs of poly(His)-tagged SRPK2 and core protein dimer were overexpressed in BL21(DE3)pLysS in the same conditions as mentioned above. Cell pellets with overexpressed recombinant proteins were homogenized and lysed in lysis buffer (20 mM Tris, pH 7.4, 350 mM NaCl, 10 mM imidazole, 10% glycerol) by sonication. Proteins were purified by affinity chromatography using Ni-NTA resin. After extensive wash, bound proteins were eluted using the buffer containing the same ingredients but 200 mM imidazole. Eluted proteins were dialyzed against dialysis buffer (20 mM Tris, pH 7.4, 350 mM NaCl, 10% glycerol, 10 mM DTT), then concentrated and subjected to size-exclusion chromatography. Fractions were collected and subjected to SDS-PAGE analysis. Fractions containing the purified proteins were then concentrated and frozen at -80°C. His-tagged CpWT (apo-capsid) was overexpressed in BL21(DE3) pLysS and purified using Ni-NTA chromatography. In brief, collected cells were lysed in lysis buffer (20 mM Tris, pH 7.4, 350 mM NaCl, 10 mM imidazole, 10% glycerol, 6 M urea) by sonication. After extensive washes, bound protein was eluted with elution buffer (20 mM Tris, pH 7.4, 350 mM NaCl, 200 mM imidazole, 10% glycerol, 6 M urea). The eluted protein was then refolded and allowed to assemble into capsids by dialyzing against the refolding buffer (20 mM Tris, pH 7.4, 350 mM NaCl, 200 mM imidazole, 10% glycerol) in a stepwise manner. The capsids were further purified using size-exclusion chromatography (Hiload 16/60 Superdex 200pg). Fractions were collected and subjected to SDS-PAGE analysis. Fractions containing the purified capsids were then concentrated and frozen at -80°C.

### GST-pull down assays

500 nM GST-CpY132A constructs were immobilized on glutathione resin in pull down buffer (20 mM Tris, pH7.4, 150 mM NaCl, 5% glycerol, 1 mM DTT, 1 mM benzamidine, 0.5% Triton-X100) overnight at 4°C. Unbound core protein was washed away and then 1 μM prey proteins (SRPKs) were added into the resin and incubated for another 3 hours at 4°C. Resin was washed with the buffer for 3 times afterwards. Samples were analyzed by SDS-PAGE following the addition of SDS loading dye and heating of samples at 98°C for 3 mins.

### *In vitro* kinase assay

Purified wild-type SRPKs and HBV core proteins were mixed in 2 to 1 molar ratio and allowed to incubate in kinase reaction buffer (50 mM Tris, pH 7.4, 150 mM NaCl, 10 mM MgCl_2_) at room temperature. The kinase activity was initiated by adding 50 μM unlabeled ATP and 0.5 μCi of [γ -32P] ATP at room temperature. Samples were aliquoted at the corresponding time points and the reactions were quenched by SDS loading buffer, followed by heating of the samples at 98°C for 2 mins. Samples were resolved by SDS-PAGE and visualized by Coomassie Brilliant Blue staining. The gel was dried on Whatman filter paper (GE Healthcare) and then subjected to autoradiography using medical X-ray films (Fujifilm).

### Microscale thermophoresis (MST)

Monolith His-Tag labeling kit (RED-tris-NTA) was used to label 200 nM of N-terminally His-tagged wild-type SRPK1/2 as target proteins according to the manufacturer’s protocol. Labeled proteins were mixed with the 2-fold serial diluted ligand (GST-CpY132A), concentration from 20 μM to 0.61 nM. Protein complexes were loaded into Monolith NT.115 capillaries and measured using Monolith NT.115 and MO. Control software at room temperature (LED/excitation power setting medium, MST power setting 40%). MST trace data was analyzed by MO. Affinity Analysis software (version 2.2.5, NanoTemper Technologies) at the standard MST-on time of 5 seconds. Protein concentrations were quantified and normalized by silver staining (Pierce).

### Chemical crosslinking

Experimental design is referred to the previous study [25]. Surface-exposed cysteine in SRPK2ΔS1 (C131 and C223) and that in CpY132A (C48 and C185) were mutated to serine to reduce non-specificity. In the surface cysteine-free background, a further mutation in SRPK2(K648C) and 4 independent mutations on CpY132A (T147C, S157C, S170C and S178C) were generated. In each reaction, 2 μM kinase was incubated with 2 μM of one of the core protein mutants in crosslinking buffer (20 mM Tris, pH7.4, 250 mM NaCl, 5 mM MgCl_2_, 5% glycerol) for 15 mins at ambient temperature. Each reaction was divided into equal-volume aliquots and supplemented with different concentration of ATP from 0 μM to 12 μM to allow phosphorylation to occur. After 8 mins, phosphorylation was stopped by adding 5 mM EDTA. All reactions were then placed on ice to allow cooling down. Then, 0.01 mM 1,4-bismaleimidobutane (BMB) (Pierce) was added into the reactions and incubated for 8 mins on ice. Controls were performed accordingly: protein alone with BMB in the reaction and complex without BMB in the reaction. Reducing SDS loading buffer was added to the reactions while non-reducing dye was added to the controls. Finally, samples were resolved by SDS-PAGE and visualized by Coomassie blue stain. The crosslinked products were further confirmed by western blot using anti-His-tag antibody (Santa Cruz).

### Processivity assay

Processivity assay was performed as reported before [26]. 1) No Trap assay. One micromolar SRPK2 was incubated with 500 nM CpY132A in kinase assay buffer (50 mM Tris pH7.4, 150 mM NaCl, 10 mM MgCl_2_) at ambient temperature for 15 mins. 2) Start-Trap assay. A kinase-dead SRPK2 mutant was used as trap. 1 μM SRPK2 was incubated with 500 nM CpY132A in kinase assay buffer at ambient temperature. 40 μM trap was added as the reactions started. 3) Trap-Start assay. 40 μM trap and 500 nM CpY132A was incubated in kinase assay buffer at ambient temperature. One micromolar SRPK2 was then introduced into the mixture at the start of reactions. All reactions were initiated by adding 50 μM unlabeled ATP and 0.5 μCi of [g-32P] ATP at 25°C. Samples were aliquoted at the corresponding time points and quenched by SDS loading dye, followed by heating of the samples at 98°C for 2 mins. Samples were analyzed by SDS-PAGE. The gel was dried on Whatman filter paper (GE Healthcare) and then subjected to autoradiography using medical X-ray films (Fujifilm).

### HBV capsid assembly analysis by size-exclusion chromatography (SEC)

HBV capsid assembly assay was performed following the previous study [15]. Three experiments were set up: 1) Purified CpWT in disassembly buffer was dialyzed against reassembly buffer to allow capsid formation. 2) Purified CpWT and SRPK2ΔS1 were mixed in 1 to 1 molar ratio in disassembly buffer. After removing GuHCl by dialysis, SRPK2ΔS1/CpWT complex was obtained for further purification using SEC (analytical Superdex 200 10/300 GL column (Cytiva)). 3) SRPK2ΔS1/CpWT complex was supplemented with 5 mM MgCl_2_ and 0.1 mM ATP. All the above reactions were subject to SEC for analysis. Similar experiments were performed for assay using SRPK2ΔS1DM instead.

### Sample preparation for cryo-EM

CpWT was overexpressed in BL21(DE3)pLysS and purified in buffer containing 6M urea using Ni-NTA chromatography as described above. Protein was refolded and allowed to assemble into apo-capsid by removing urea via dialysis. The apo-capid was further purified using size-exclusion chromatography. Glycerol in the sample buffer was removed by dialysis before cryo-EM.

SRPK2ΔS1/CpWT capsid complex was purified as described previously [15]. N-terminally His-tagged SRPK2ΔS1 and N-terminally His-tagged CpWT were overexpressed in BL21(DE3)pLysS. Cell pellet with overexpressed His-SRPK2ΔS1 was homogenized in lysis buffer A (20 mM Tris, pH 7.4, 350 mM NaCl, 10 mM imidazole, 10% glycerol) while cell pellet with overexpressed His-CpWT was homogenized in lysis buffer B (1.5 M GuHCl, 20 mM Tris, pH 7.4, 500 mM LiCl, 10 mM imidazole, 10% glycerol) with addition of RNase A. After cell lysis by sonication, two proteins were purified separately using nickel column. After extensive wash, bound proteins were eluted using buffer A or B, containing 200 mM imidazole instead. Different fractions (flow-through, wash, elution) were collected and analyzed by SDS-PAGE. Gel was stained by Coomassie Blue. Eluted proteins were dialyzed against disassembly buffer (1.5 M GuHCl, 20 mM Tris, pH 7.4, 350 mM NaCl, 10% glycerol, 10 mM DTT). To prepare SRPK2ΔS1/CpWT capsid complex, equimolar amounts of His-CpWT and His-SRPK2ΔS1 were mixed and incubated at 4°C. The mixture was then dialyzed against the reassembly buffer (20 mM Tris, pH 7.4, 350 mM NaCl, 10% glycerol, 10 mM DTT) at 4°C. Complex was further purified by size-exclusion chromatography (Hiload 16/60 Superdex 200pg). Peak fraction containing SRPK2ΔS1/HBV capsid complex was concentrated and frozen at -80°C. Protein complex was dialyzed to remove glycerol before cryo-electron microscopy.

### Immunogold labelling and negative stain-transmission electron microscopy

Carbon film on 400 mesh grids (EMR) were glow discharged for 60 seconds at 30 mA current to increase the hydrophilicity. Twenty-five microliters of purified SRPK2ΔS1/CpWT capsid complex was diluted to 0.05 mg/ml and floated on the mesh grids, and incubated for 2 minutes at room temperature. Then, the sample grids were washed in 25 μl ddH_2_O for 1 minute and then incubated in 25 μl blocking buffer (0.3% BSA in TBS-T) for 30 minutes in a humidified chamber. After that, sample grids were incubated in anti-SRPK antibody (Santa Cruz) (diluted in 1:100 ratio in blocking buffer) for 1 hour and then washed 3 times in 25 μl washing buffer (0.03% BSA in TBST) for 3 minutes. After the washing steps, sample grids were incubated in 25 μl of the 10 nm gold goat anti-mouse IgG (Abcam) (diluted in 1:20 ratio in blocking buffer) for 1 hour in a humidified chamber. The sample grids were then washed 3 times in 25 μl TBST for 3 minutes, followed by 3 washes in 25 μl ddH_2_O for 10 seconds each. Next, the sample grid was stained by 25 μl of 2% uranyl acetate for 10 seconds and then transfered to another 25 μl of 2% uranyl acetate for 30 seconds. The grids were allowed to be air dried under room temperature for 30 minutes. Sample grids were then examined under TEM (Hitachi- H7650) using AMT imaging system.

### Electron microscopy data acquisition

Three μl of purified sample (1 mg/mL) was applied to a glow-discharged holey carbon grid (Quantifoil, R 1.2/1.3, 400 mesh). The grid was blotted with a Vitrobot Mark IV (ThermoFisher) at 4°C and 100% relative humidity, followed by plunge freezing in liquid ethane at liquid nitrogen temperature. Images were acquired with a Talos F200C electron microscope (ThermoFisher) operated at 200 kV and equipped with a Falcon 3EC direct electron detector (ThermoFisher). The data was recorded in electron counting mode at a nominal magnification of 50,000x, corresponding to a calibrated pixel size of 0.969 Å. The total electron exposure was ~50 e^-^/Å^2^ on specimen, fractionated in 40 frames, with a defocus range between -0.5 and -3.0μM.

### Image processing

All the following image-processing steps were performed in RELION-3.1.2 [52]. Firstly, all data were binned (bin = 2; pixel size 1.938 Å). Contrast transfer function parameters of each micrographs were estimated by using CTFFIND 4.1 [53]. Particles were automatically picked and extracted from 824 micrographs. After reference-free 2D classification to remove bad quality particles, 21,604 particles were selected for 3D classification. For 3D classification, an 8.1 Å model of HBV capsid (EMD-3015) [54] was low-pass filtered to 60 Å and used as an initial model for 3D classification with icosahedral symmetry applied. 6,266 particles were subjected to further 3D refinement, generating a final map with icosahedral symmetry and resolution at 4.6 Å. The unmasked resolutions of the apo-capsid and the SRPK2/HBV capsid complex are 5.03 Å and 4.64 Å, respectively. As for the focused refinement of the sub-particle on 2-fold vertex. A total number of 6,266 refined particles from the final 3D refinement were symmetry-expanded with icosahedral symmetry. A reference mask covering the sub-particle on one of the 2-fold vertex was created from the 3D icosahedral reconstruction using “volume eraser” in UCSF Chimera, followed by processing in “mask creation” on RELION with a lowpass filter of 15 Å. Expanded particles of 375,960 were subtracted by the reference mask without centering. Subtracted particle images were subjected to a masked 3D classification in which refined orientations from the previous consensus refinement of the whole complex were input. The classification was operated with C1 symmetry, regularization parameter T = 40 and local angular search. A class of 58,259 sub-particles was chosen for the subsequent masked 3D auto-refinement with C1 symmetry applied. Map of SRPK2ΔS1/CpWT capsid complex at 2-fold vertex was reconstructed at 11 Å. Resolution was determined by gold-standard FSC = 0.143 criterion [55]. Final maps were sharpened and filtered locally by local-resolution estimation in RELION. Map segmentation was performed using UCSF Chimera. Crystal structure of SRPK2 (PDB: 5MYV) was fitted in the final maps using “Fit in map” in UCSF Chimera.

### CRISPR / Cas9-mediated gene knockout of SRPK2 in HepG2

To perform the SRPK2 gene knockout, two guide RNAs were designed, sequence of gRNA1 is 5’- TCGTAATATGCCTCTGTCGGCCAC- 3’ while sequence of gRNA2 is 5’- CGAGTAACTGCTGAAGTTCTGCCAC- 3’, targeting at nucleotides 100,962–100,981 and nucleotides 101,599–101,618 in SRPK2 gene respectively. These two gRNAs were cloned into PX459 according to the protocol from Dr. Feng Zhang’s lab [56]. HepG2 cells, cultured in EMEM containing 10% FBS, were co-transfected with the aforementioned PX459 with gRNAs cloned in, using Lipofectamine 2000 (Invitrogen). After 48 hours transfection, final concentration of 4 μg/mL was added into the medium and wait for another 48 hours. Selected cells were recovered in fresh EMEM followed by a 96-well serial dilution. Single-cell colonies were isolated and cultured. Western blot experiment and DNA sequencing was performed to check the knockout in the corresponding clones. Anti-SRPK2 antibody (BD Transduction Laboratories) and anti-beta-tubulin antibody (GenScipt) were used. Successful clones were frozen and stored in liquid nitrogen.

### Transfection of HepG2 cell lines and pull-down assays

Both control HepG2 and HepG2(K2KO) cells were cultured in EMEM (ATCC), supplemented with 5% CO_2_. Cells were seeded in 12 well-plate and transfected with pcDNA-myc-his-Cp using EndoFectin HepG2 (Genecopoeia) for 24 hours, and one well of the HepG2(K2KO) cells was treated with 3 μM SPHINX 31. Transfected cells were collected and lysed in the lysis buffer (20 mM Tris, pH 7.4, 150 mM NaCl, 1% NP-40, supplemented with 1 mM Benzamidine and phosphatase inhibitor cocktail (Sigma) and protease inhibitor cocktail (MedChemExpress)). Cell lysates were incubated with Ni-NTA resin at 4°C for 1 hour. Beads were washed by the lysis buffer and centrifuged. SDS loading dye was added to the samples after removal of the supernatant. Samples were boiled at 98°C and analyzed by western blot using anti-CP antibody (C-1, Abcam), anti-phospho-SR antibody (mAb 104), anti-phosphoserine antibody (Santa Cruz), anti-β-tubulin antibody (GenScript), and anti-SRPK2 antibody (BD Transduction Laboratories).

For the *in vitro* GST pull-down assay using cell lysates, both HepG2 and HepG2(K2KO) cells were lysed using lysis buffer (20 mM Tris, pH 7.4, 150 mM NaCl, 1% NP-40, supplemented with 1 mM Benzamidine and phosphatase inhibitor cocktail (Sigma) and protease inhibitor cocktail (MedChemExpress)). Cell lysates were incubated with 1 μM purified recombinant GST-CpWT and GST-glutathione resin (GenScript) at 4°C for 3 hours. Beads were washed by the lysis buffer and pelleted by centrifugation. SDS dye was added to the samples after removal of the supernatant. Samples were boiled at 98°C and analyzed by western blot using anti-phosphoserine antibody (Santa Cruz), anti-GST antibody (Santa Cruz), anti-β-tubulin antibody (GenScript), and anti-SRPK2 antibody (BD Transduction Laboratories).

### Statistical analysis

One-way ANOVA or Student’s t-test was used to determine statistical significance. P-value < 0.05 is considered as statistically significant.

## Supporting information

S1 Fig Essential regions of SRPK2 for binding with Cp. (A) Domain organization of SRPK2 constructs used for the *in vitro* GST pull-down assay. SRPK2ΔN, SRPK2ΔNS1, and SRPK2ΔS1 represent different truncation constructs of SRPK2; SRPK2DM is a docking groove mutant with 4 critical amino acid residues mutated to alanine. (B) *In vitro* GST pull-down assay was performed using GST-tagged Cp and His-tagged SRPK2 constructs. Results were analyzed by SDS-PAGE. Spacer region and N-terminal proline-rich motif are dispensable to the binding between SRPK2 and Cp while the docking groove is significant to the binding of SRPK2 and Cp.
(TIF)

S2 Fig Mapping the binding region(s) on Cp for SRPK2. (A) Arginine-rich domain (ARD) serves as a binding motif for SRPK2. *In vitro* GST pull-down assay was performed using SRPK2WT, GST-CpY132A, and GST-Cp149Y132A, of which the ARD was truncated. Reactions were analyzed by SDS-PAGE. Free GST was used as a control. Absence of ARD in Cp abolished the binding of SRPK2 (B) Schematic diagram shows the sequences of the ARD in CpY132A. Mutational constructs generated for the study of protein-protein interaction are shown. (C) Arginine-rich motifs in ARD of Cp serve as binding motifs for SRPK2. GST-tagged mutational Cp constructs and His-SRPK2WT were used in the *in vitro* GST pull-down assay, free GST as a control. Results were analyzed by SDS-PAGE. Mutations of arginine-rich motif 1 in ARD of Cp greatly weakened the binding of SRPK2. (D) Mutations of arginine-rich motifs in ARD of Cp do not have distinct effects on SRPK2 phosphorylation. *In vitro* kinase assay using [γ-32P] ATP, SRPK2WT and mutational Cp constructs was performed. Samples were analyzed by SDS-PAGE, followed by autoradiography. The phosphorylation content of GST-tagged CpY132A M1-M4 were similar.
(TIF)

S3 Fig Control experiment verifying the specificity of the mutation-directed crosslinking reaction. (A) Crystal structure of SRPK1 in complex with a 9-mer peptide (PDB ID: 1WBP). The binding interface of the docking groove of SRPK1 and the 9-mer peptide that mimics a substrate docking motif is shown (right). (B) Crystal structure of SRPK1 in complex with SRSF1 (PDB ID: 3BEG). The binding interface of the docking groove of SRPK1 and the N’-RS1 of SRSF1 is shown (right). (C) CpY132A(surface C to S), with all surface-exposed cysteine residues mutated, was used as a control for the chemical crosslinking. Reactions were performed in the presence of increasing concentrations of ATP. The molar ratio of ATP to Cp is indicated. SDS-PAGE analysis showed that CpY132A(surface C to S) did not non-specifically crosslink with SRPK2ΔS1(K648C).
(TIF)

S4 Fig Western blots of chemical crosslinking experiments. Crosslinked complexes of His-SRPK2 and different His-CpY132A mutants formed after the addition of BMB and ATP were revealed by western blotting using anti-His tag antibody. Cp or SRPK2 alone with BMB, and SRPK2 and Cp without BMB were included as controls.
(TIF)

S5 Fig Immunogold labelling of SRPK2ΔS1/CpWT capsid complex. Immunogold-labelled protein complex was examined under negative-stain TEM. Anti-His colloidal gold labelling was observed on the capsid particles, indicating that His-SRPK2ΔS1 was presented on the surface of the capsid. The scale bar is 50 nm.
(TIF)

S6 Fig Purification of SRPK2ΔS1/CpWT capsid complex for cryo-EM. (A) Recombinant His-SRPK2ΔS1 and His-CpWT were purified via Ni-NTA affinity chromatography. Proteins were dialyzed against the disassembly buffer prior to complex formation. The proteins were mixed and incubated, followed dialysis against the reassembly buffer. Further purification was done using size-exclusion chromatography. Peak fractions were analyzed by SDS-PAGE (upper panel). Fractions of peak I containing the SRPK2ΔS1/CpWT capsid complex were collected and concentrated (lower panel). (B) The concentrated protein from peak I was negatively stained and examined by TEM. The scale bar is 100 nm.
(TIF)

S7 Fig Overall cryo-EM structures of CpWT capsid and the complex with SRPK2ΔS1. (A-B) 3D reconstruction and the cutaway views of (A) the apo-capsid and (B) the complex of SRPK2ΔS1 and CpWT capsid. Density maps are colored according to the local resolutions. 2-fold, 3-fold, and 5-fold axes are denoted by an oval, a triangle, and a pentagon respectively. The scale bars represent 10 nm. The corresponding FSC curves are shown on the right. (C) The crystal structure of the HBV core protein (PDB: 1QGT) is fitted into the density maps of the apo-capsid and SRPK2ΔS1/CpWT. The N-termini of four core protein monomers were indicated by red arrows. Extra densities attributed by the His-tags of CpWT are indicated. No extra unaccounted density is observed. (D) Overall cryo-EM 3D reconstruction and resolution assessment of the focus-refined sub-particle. The 3D reconstruction is colored by the local resolution. Scale bar represents 5 nm. The FSC curve is shown on the right.
(TIF)

S8 Fig Processing of cryo-EM images of CpWT capsid and the complex with SRPK2ΔS1. (A) Representative micrograph of SRPK2ΔS1/CpWT capsid complex with a scale bar of 100 nm (left). White arrowheads indicate the protruding ends on the capsid surface. Reference-free 2D class averages of particles extracted from micrographs (right). 2D class averages were divided into two groups: capsids with extra densities on surface, which are indicated by red arrowheads, and capsids without extra surface densities. Particles with extra surface densities were used for the subsequent 3D classification. The box size is 512 Å. (B) Representative micrograph of CpWT capsid with a scale bar of 100 nm (left). Reference-free 2D class averages of particles extracted from micrographs (right). The box size is 480 Å.
(TIF)

S9 Fig 3D classification of I symmetry-expanded subparticles. The 3D classification of subparticles after symmetry-expansion and particle subtraction. Class #1–5 are labelled in grey, yellow, cyan, purple, and pink respectively. The population of particles is indicated respectively. Class 5 was chosen for the subsequent masked 3D auto-refinement. The scale bar is 10 nm.
(TIF)

S10 Fig SRPK1 and SRPK2 do not have additive effect on CpWT capsid phosphorylation. (A) Purification of His-CpWT. His-CpWT were purified using Ni-NTA affinity chromatography in the presence 6M urea. Samples were resolved by SDS-PAGE. (B) GuHCl was removed by dialysis to allow His-CpWT to assemble into capsids. The capsids were further purified by gel filtration. Protein collected from the peak fractions was analyzed by SDS-PAGE. (C) *In vitro* radioactive kinase assay was performed using the CpWT capids in the presence of SRPK1WT or SRPK2WT or both kinases. Samples were analyzed by SDS-PAGE and autoradiography. Presence of both SRPK1 and SRPK2 did not result in more phosphorylated Cp product.
(TIF)

S11 Fig Generation of SRPK2-knockout HepG2 cell line. Genomic DNA from the cell lines was extracted for sequencing. The position where the DNA sequence of SRPK2 was altered by CRISPR-Cas9 is indicated (upper panels). Heterozygous sequencing results indicated that the knockout was biallelic and the altered DNA sequence in two alleles are labelled in red and blue respectively (bottom panels).
(TIF)

S12 Fig SRPK2 is important for the phosphorylation Cp. The effect of SRPK2 knockout on the phosphorylation of core protein in HepG2 and HepG2(K2KO) cell lines. Lysates of control HepG2 and HepG2(K2KO) cells were incubated with recombinant GST-CpWT. GST-CpWT was then isolated using glutathione resins and analyzed by western blot using anti-phosphoserine antibody. The phosphorylation of GST-CpWT using HepG2(K2KO) lysate was lowered when compared to that using control HepG2 cells. GST, which was phosphorylated by unknown kinase(s) in the lysates, was included as a control.
(TIF)

S13 Fig SRPK1 does not phosphorylate T162 *in vitro*. *In vitro* radioactive kinase assay was performed using SRPK1WT and the mutational Cp constructs, with six/seven alanine substitution at the phosphorylatable sites, in the presence of [^32^P]ATP. Reactions were quenched after 10 mins and analyzed by SDS-PAGE. The gel was visualized with Coomassie Blue staining and then subjected to autoradiography. Mutation of six serines to alanines abolished the phosphorylation of Cp by SRPK1.
(TIF)

S1 Table Data acquisition and processing of single-particle-analysis cryoEM. (DOCX)
