# Supplementary figures and images for: SRPK2 Mediates HBV Core Protein Phosphorylation and Capsid Assembly via Docking Interaction
Source: PLoS Pathog. 2024 Feb 7;20(2):e1011978. doi: 10.1371/journal.ppat.1011978 (PMC10878513; doi:10.1371/journal.ppat.1011978)

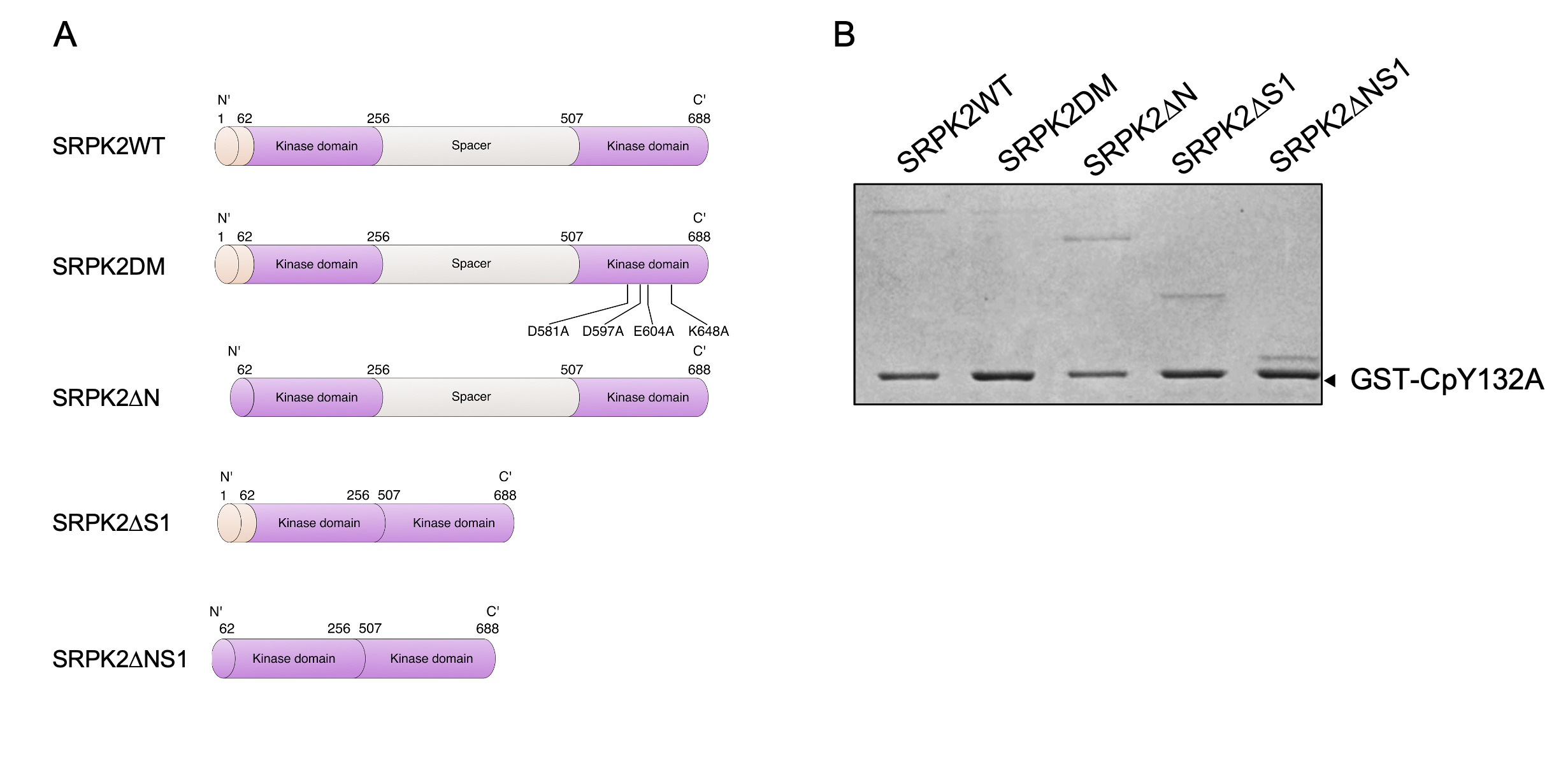

Supplement: S1 Fig — (A) Domain organization of SRPK2 constructs used for the in vitro GST pull-down assay. SRPK2ΔN, SRPK2ΔNS1, and SRPK2ΔS1 represent different truncation constructs of SRPK2; SRPK2DM is a docking groove mutant with 4 critical amino acid residues mutated to alanine. (B) In vitro GST pull-down assay was performed using GST-tagged Cp and His-tagged SRPK2 constructs. Results were analyzed by SDS-PAGE. Spacer region and N-terminal proline-rich motif are dispensable to the binding between SRPK2 and Cp while the docking groove is significant to the binding of SRPK2 and Cp. (TIF) [file ppat.1011978.s001.tif]

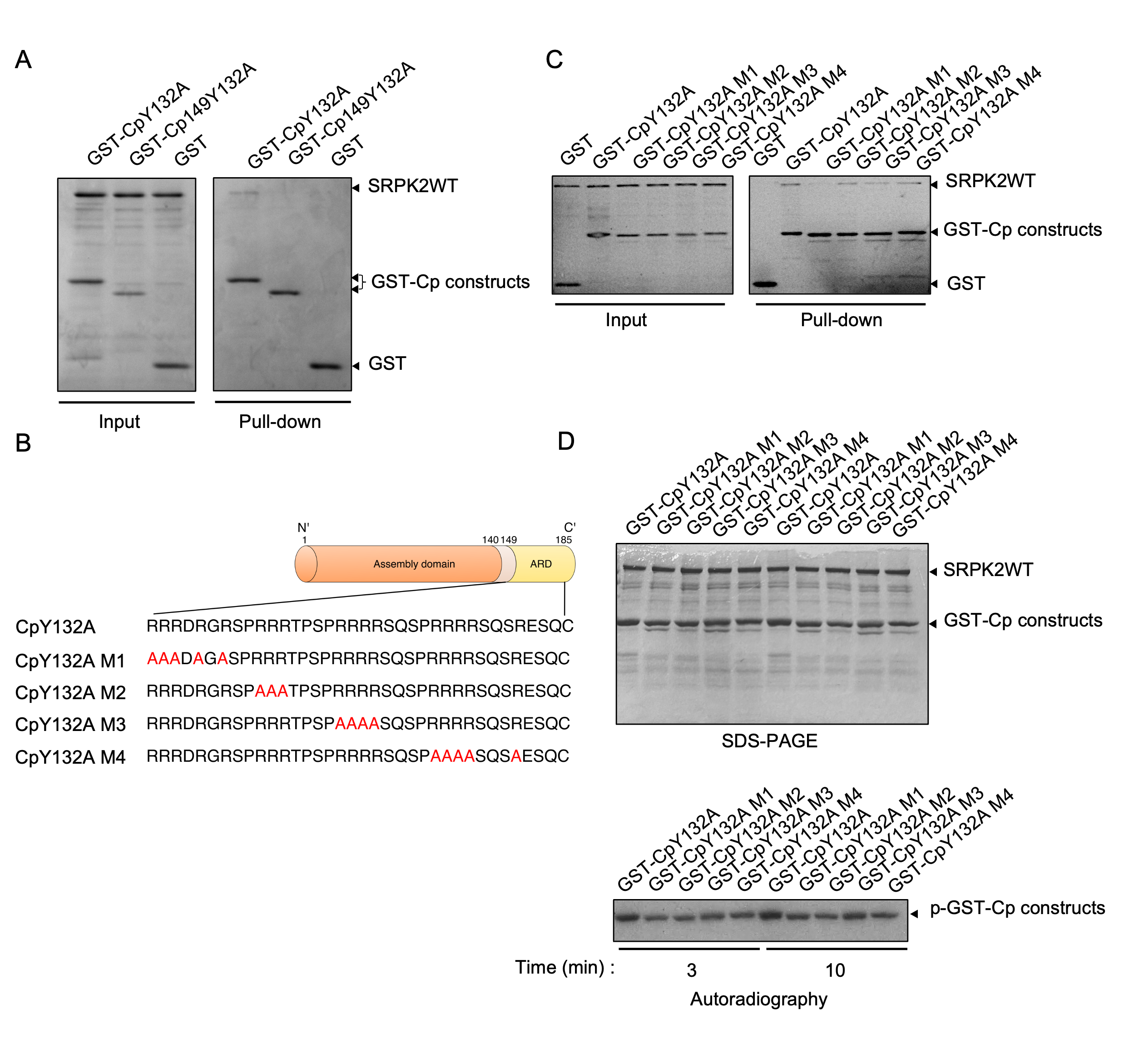

Supplement: S2 Fig — (A) Arginine-rich domain (ARD) serves as a binding motif for SRPK2. In vitro GST pull-down assay was performed using SRPK2WT, GST-CpY132A, and GST-Cp149Y132A, of which the ARD was truncated. Reactions were analyzed by SDS-PAGE. Free GST was used as a control. Absence of ARD in Cp abolished the binding of SRPK2 (B) Schematic diagram shows the sequences of the ARD in CpY132A. Mutational constructs generated for the study of protein-protein interaction are shown. (C) Arginine-rich motifs in ARD of Cp serve as binding motifs for SRPK2. GST-tagged mutational Cp constructs and His-SRPK2WT were used in the in vitro GST pull-down assay, free GST as a control. Results were analyzed by SDS-PAGE. Mutations of arginine-rich motif 1 in ARD of Cp greatly weakened the binding of SRPK2. (D) Mutations of arginine-rich motifs in ARD of Cp do not have distinct effects on SRPK2 phosphorylation. In vitro kinase assay using [γ-32P] ATP, SRPK2WT and mutational Cp constructs was performed. Samples were analyzed by SDS-PAGE, followed by autoradiography. The phosphorylation content of GST-tagged CpY132A M1-M4 were similar. (TIF) [file ppat.1011978.s002.tif]

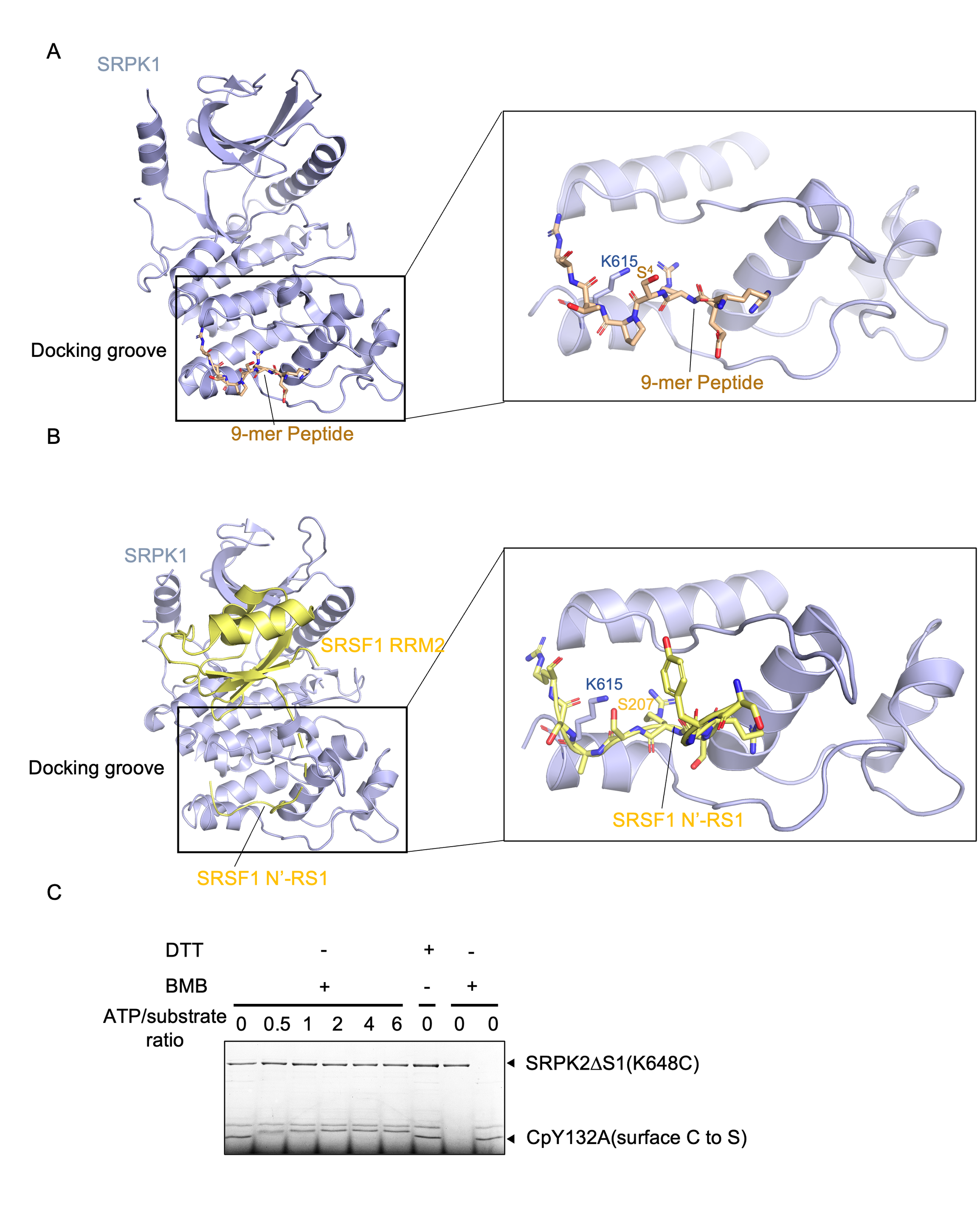

Supplement: S3 Fig — (A) Crystal structure of SRPK1 in complex with a 9-mer peptide (PDB ID: 1WBP). The binding interface of the docking groove of SRPK1 and the 9-mer peptide that mimics a substrate docking motif is shown (right). (B) Crystal structure of SRPK1 in complex with SRSF1 (PDB ID: 3BEG). The binding interface of the docking groove of SRPK1 and the N’-RS1 of SRSF1 is shown (right). (C) CpY132A(surface C to S), with all surface-exposed cysteine residues mutated, was used as a control for the chemical crosslinking. Reactions were performed in the presence of increasing concentrations of ATP. The molar ratio of ATP to Cp is indicated. SDS-PAGE analysis showed that CpY132A(surface C to S) did not non-specifically crosslink with SRPK2ΔS1(K648C). (TIF) [file ppat.1011978.s003.tif]

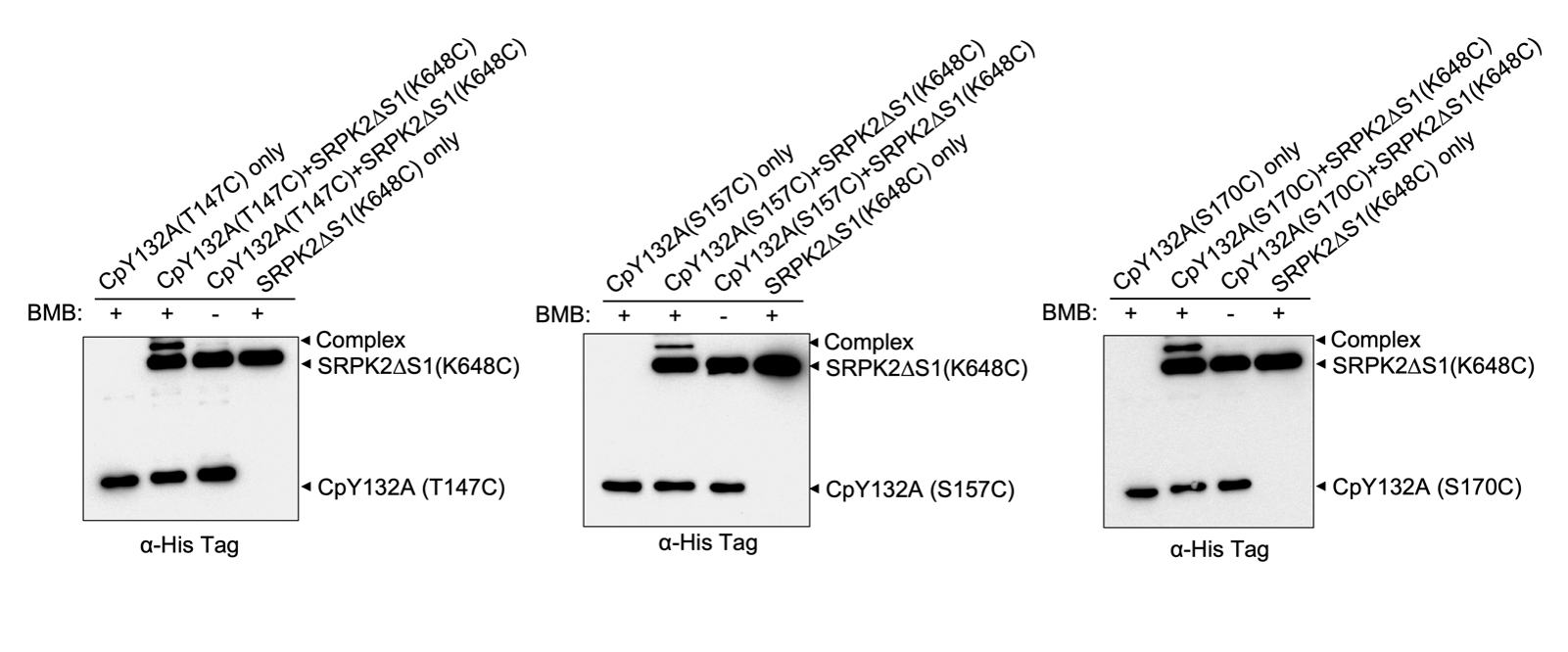

Supplement: S4 Fig — Crosslinked complexes of His-SRPK2 and different His-CpY132A mutants formed after the addition of BMB and ATP were revealed by western blotting using anti-His tag antibody. Cp or SRPK2 alone with BMB, and SRPK2 and Cp without BMB were included as controls. (TIF) [file ppat.1011978.s004.tif]

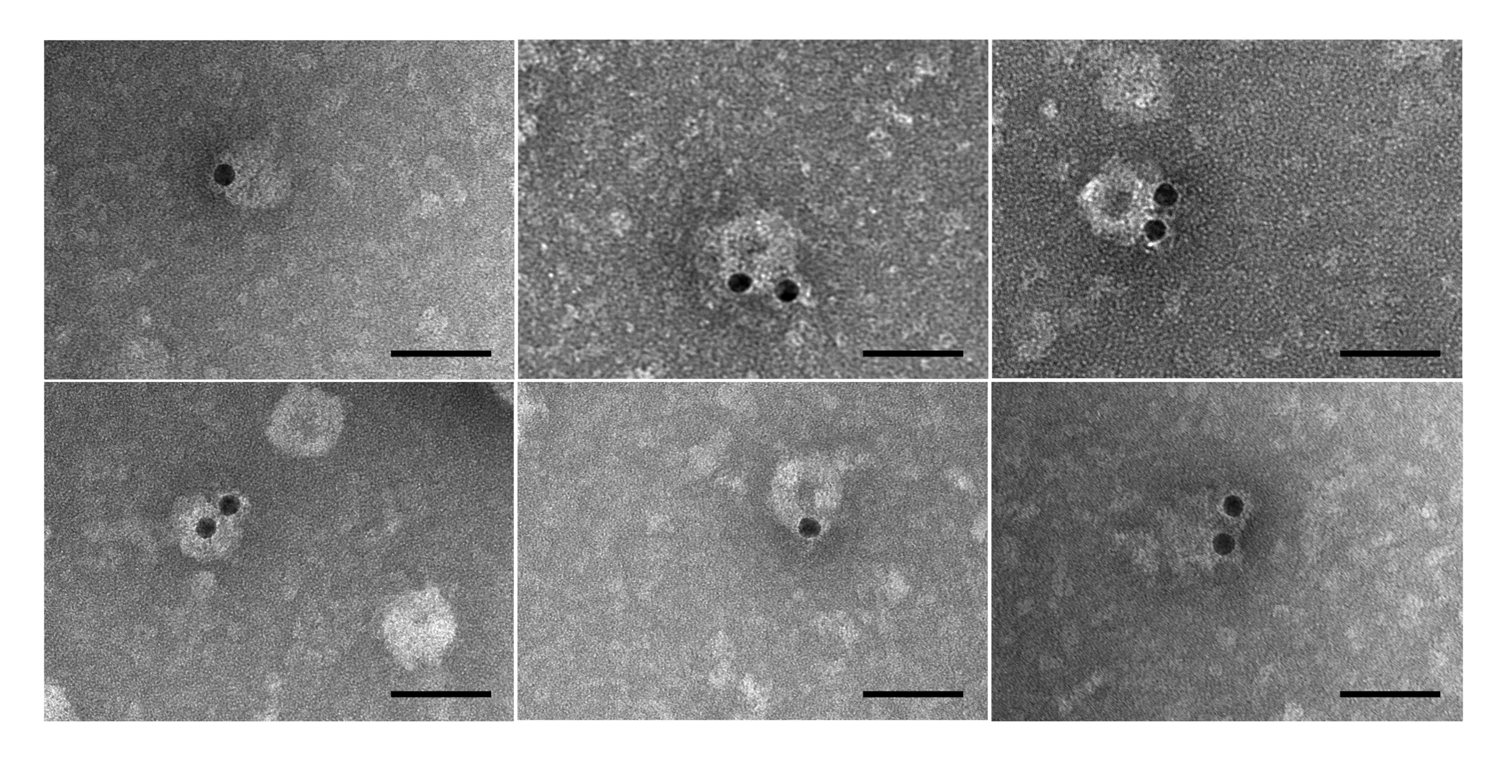

Supplement: S5 Fig — Immunogold-labelled protein complex was examined under negative-stain TEM. Anti-His colloidal gold labelling was observed on the capsid particles, indicating that His-SRPK2ΔS1 was presented on the surface of the capsid. The scale bar is 50 nm. (TIF) [file ppat.1011978.s005.tif]

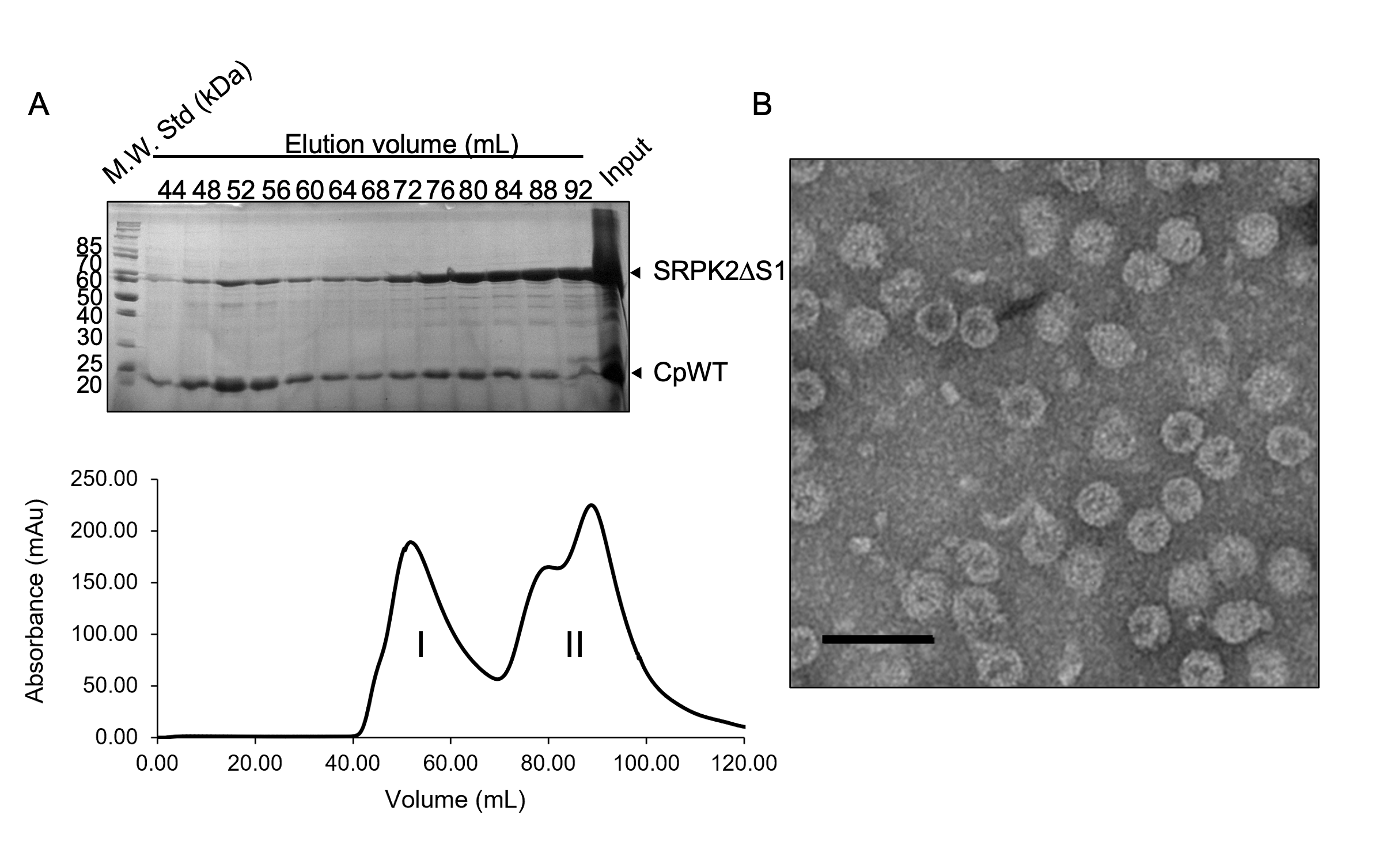

Supplement: S6 Fig — (A) Recombinant His-SRPK2ΔS1 and His-CpWT were purified via Ni-NTA affinity chromatography. Proteins were dialyzed against the disassembly buffer prior to complex formation. The proteins were mixed and incubated, followed dialysis against the reassembly buffer. Further purification was done using size-exclusion chromatography. Peak fractions were analyzed by SDS-PAGE (upper panel). Fractions of peak I containing the SRPK2ΔS1/CpWT capsid complex were collected and concentrated (lower panel). (B) The concentrated protein from peak I was negatively stained and examined by TEM. The scale bar is 100 nm. (TIF) [file ppat.1011978.s006.tif]

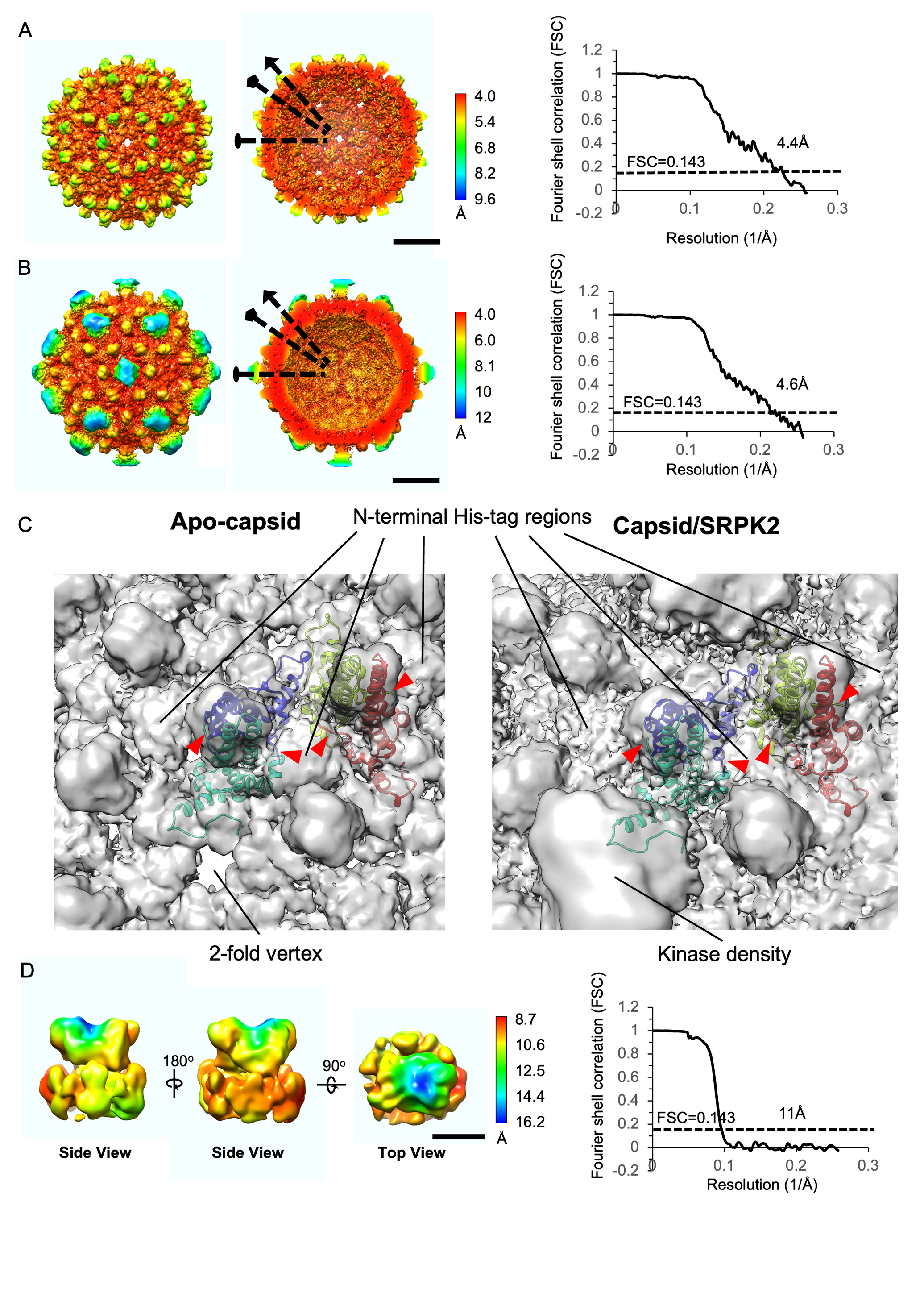

Supplement: S7 Fig — (A-B) 3D reconstruction and the cutaway views of (A) the apo-capsid and (B) the complex of SRPK2ΔS1 and CpWT capsid. Density maps are colored according to the local resolutions. 2-fold, 3-fold, and 5-fold axes are denoted by an oval, a triangle, and a pentagon respectively. The scale bars represent 10 nm. The corresponding FSC curves are shown on the right. (C) The crystal structure of the HBV core protein (PDB: 1QGT) is fitted into the density maps of the apo-capsid and SRPK2ΔS1/CpWT. The N-termini of four core protein monomers were indicated by red arrows. Extra densities attributed by the His-tags of CpWT are indicated. No extra unaccounted density is observed. (D) Overall cryo-EM 3D reconstruction and resolution assessment of the focus-refined sub-particle. The 3D reconstruction is colored by the local resolution. Scale bar represents 5 nm. The FSC curve is shown on the right. (TIF) [file ppat.1011978.s007.tif]

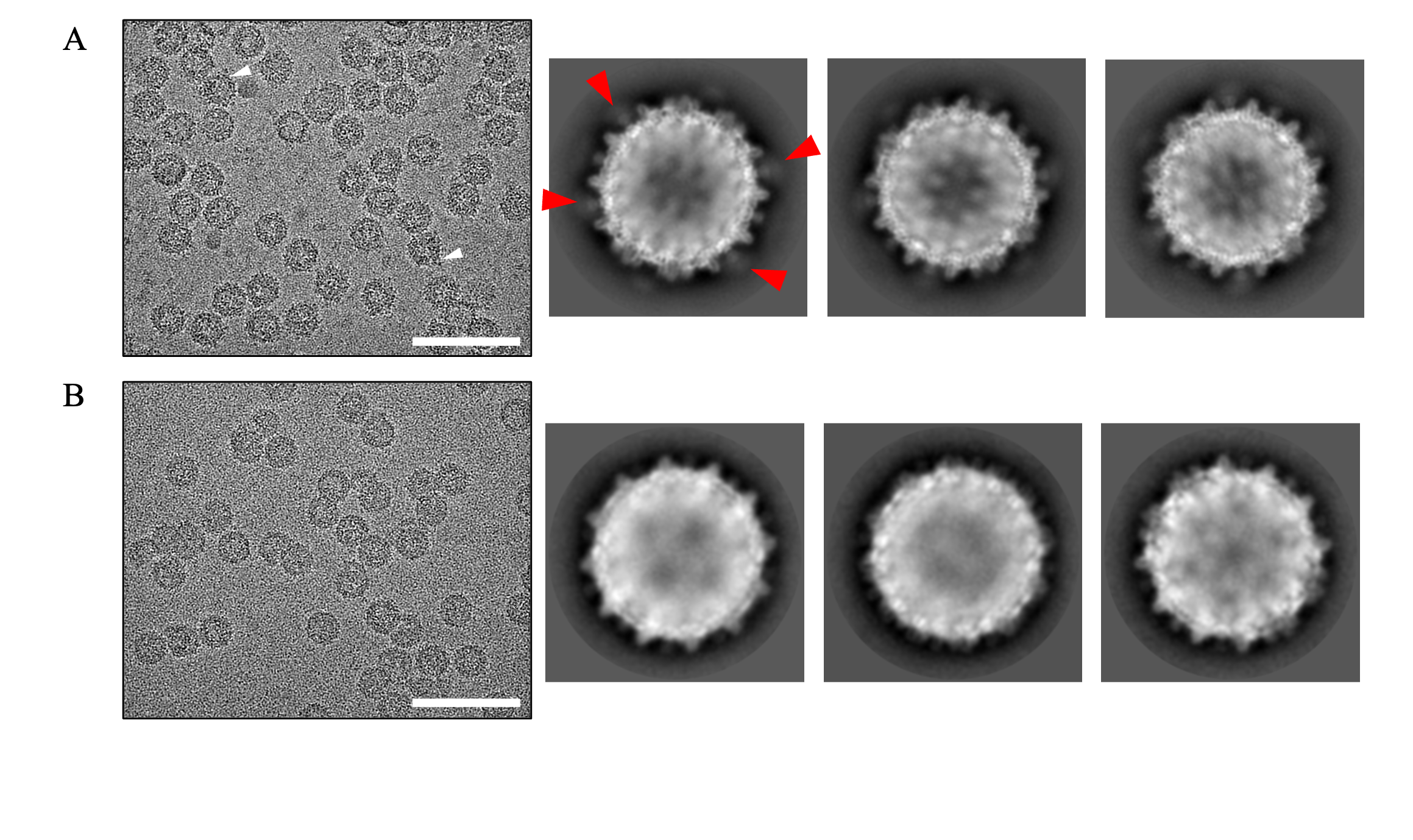

Supplement: S8 Fig — (A) Representative micrograph of SRPK2ΔS1/CpWT capsid complex with a scale bar of 100 nm (left). White arrowheads indicate the protruding ends on the capsid surface. Reference-free 2D class averages of particles extracted from micrographs (right). 2D class averages were divided into two groups: capsids with extra densities on surface, which are indicated by red arrowheads, and capsids without extra surface densities. Particles with extra surface densities were used for the subsequent 3D classification. The box size is 512 Å. (B) Representative micrograph of CpWT capsid with a scale bar of 100 nm (left). Reference-free 2D class averages of particles extracted from micrographs (right). The box size is 480 Å. (TIF) [file ppat.1011978.s008.tif]

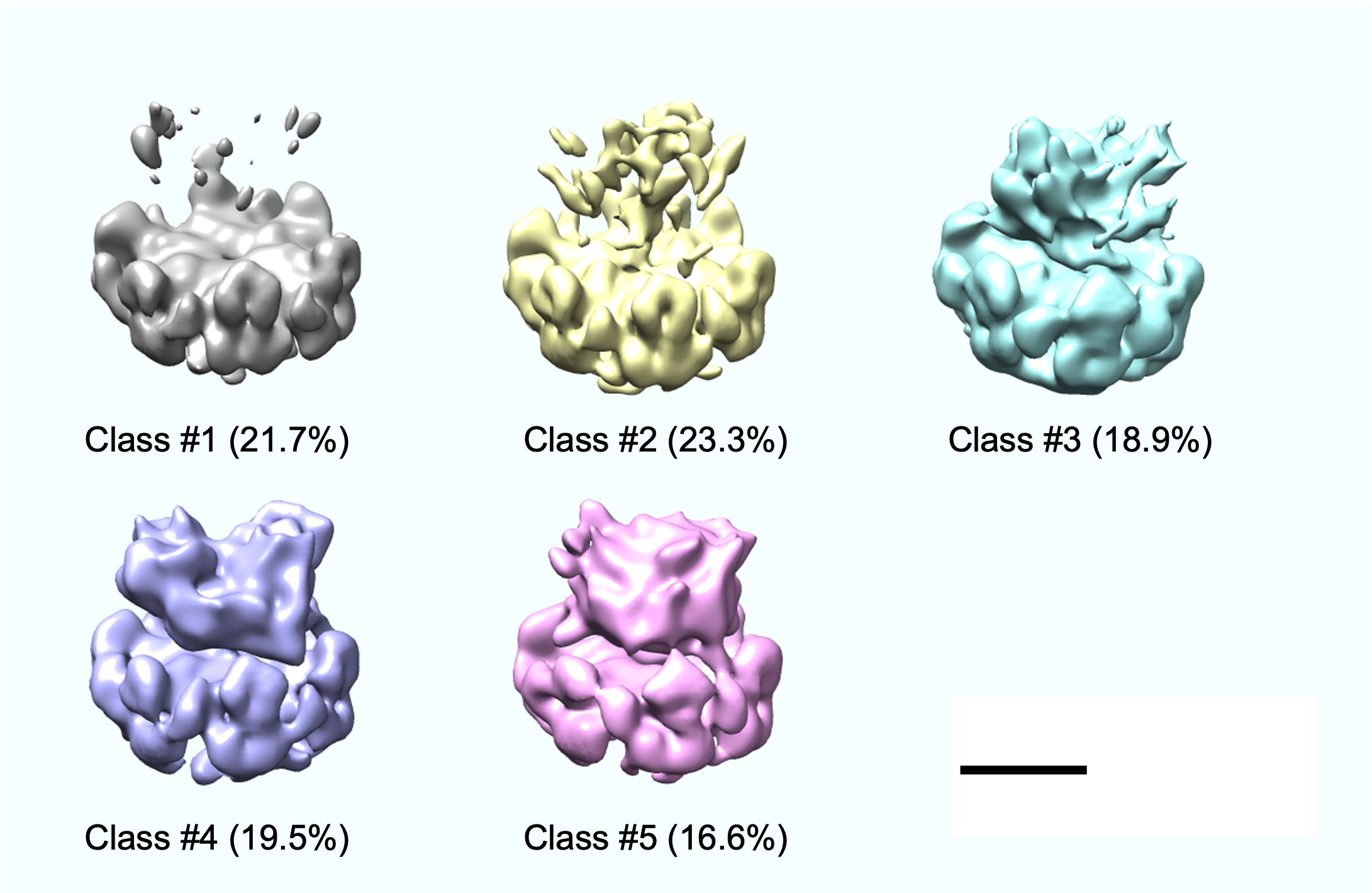

Supplement: S9 Fig — The 3D classification of subparticles after symmetry-expansion and particle subtraction. Class #1–5 are labelled in grey, yellow, cyan, purple, and pink respectively. The population of particles is indicated respectively. Class 5 was chosen for the subsequent masked 3D auto-refinement. The scale bar is 10 nm. (TIF) [file ppat.1011978.s009.tif]

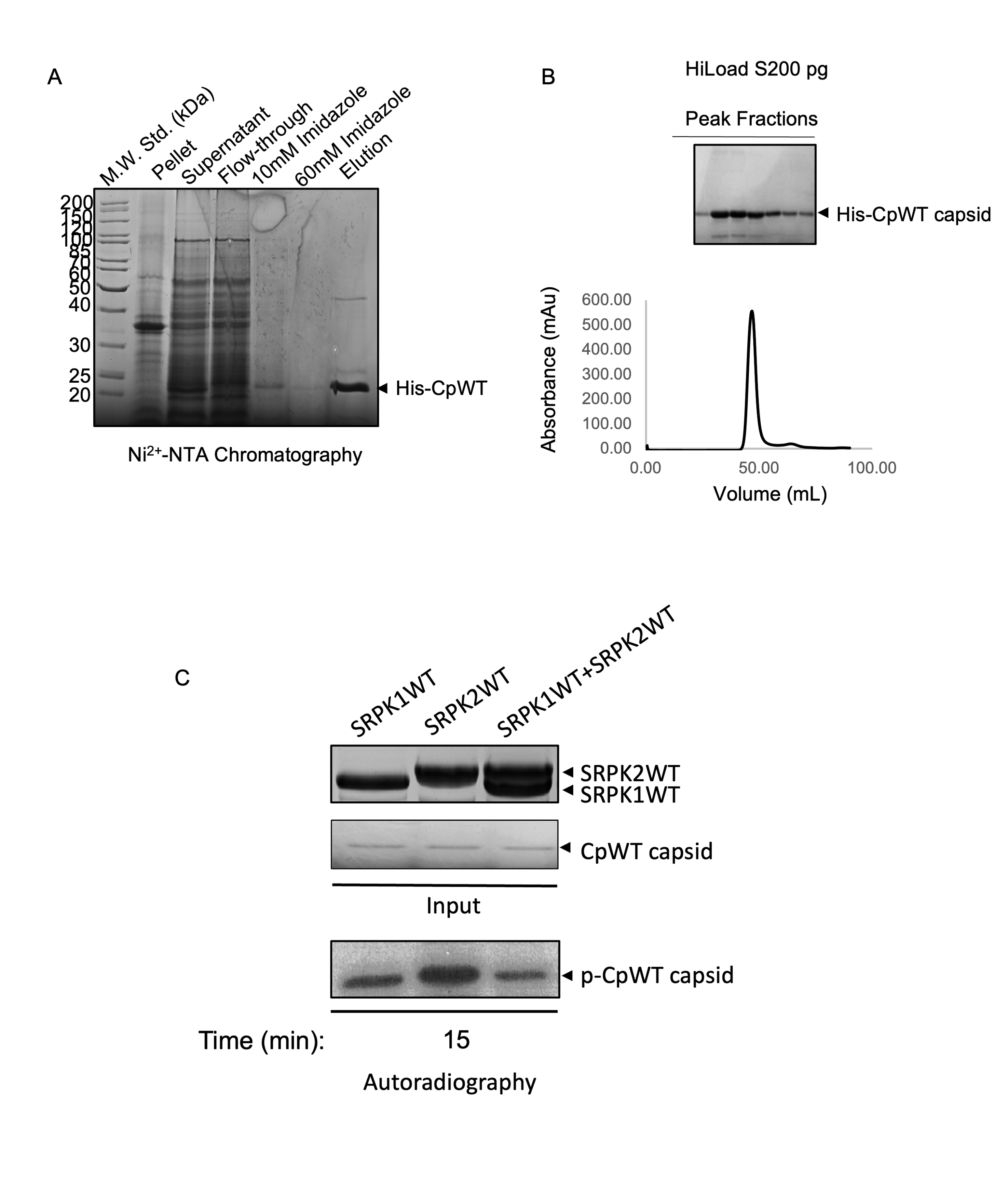

Supplement: S10 Fig — (A) Purification of His-CpWT. His-CpWT were purified using Ni-NTA affinity chromatography in the presence 6M urea. Samples were resolved by SDS-PAGE. (B) GuHCl was removed by dialysis to allow His-CpWT to assemble into capsids. The capsids were further purified by gel filtration. Protein collected from the peak fractions was analyzed by SDS-PAGE. (C) In vitro radioactive kinase assay was performed using the CpWT capids in the presence of SRPK1WT or SRPK2WT or both kinases. Samples were analyzed by SDS-PAGE and autoradiography. Presence of both SRPK1 and SRPK2 did not result in more phosphorylated Cp product. (TIF) [file ppat.1011978.s010.tif]

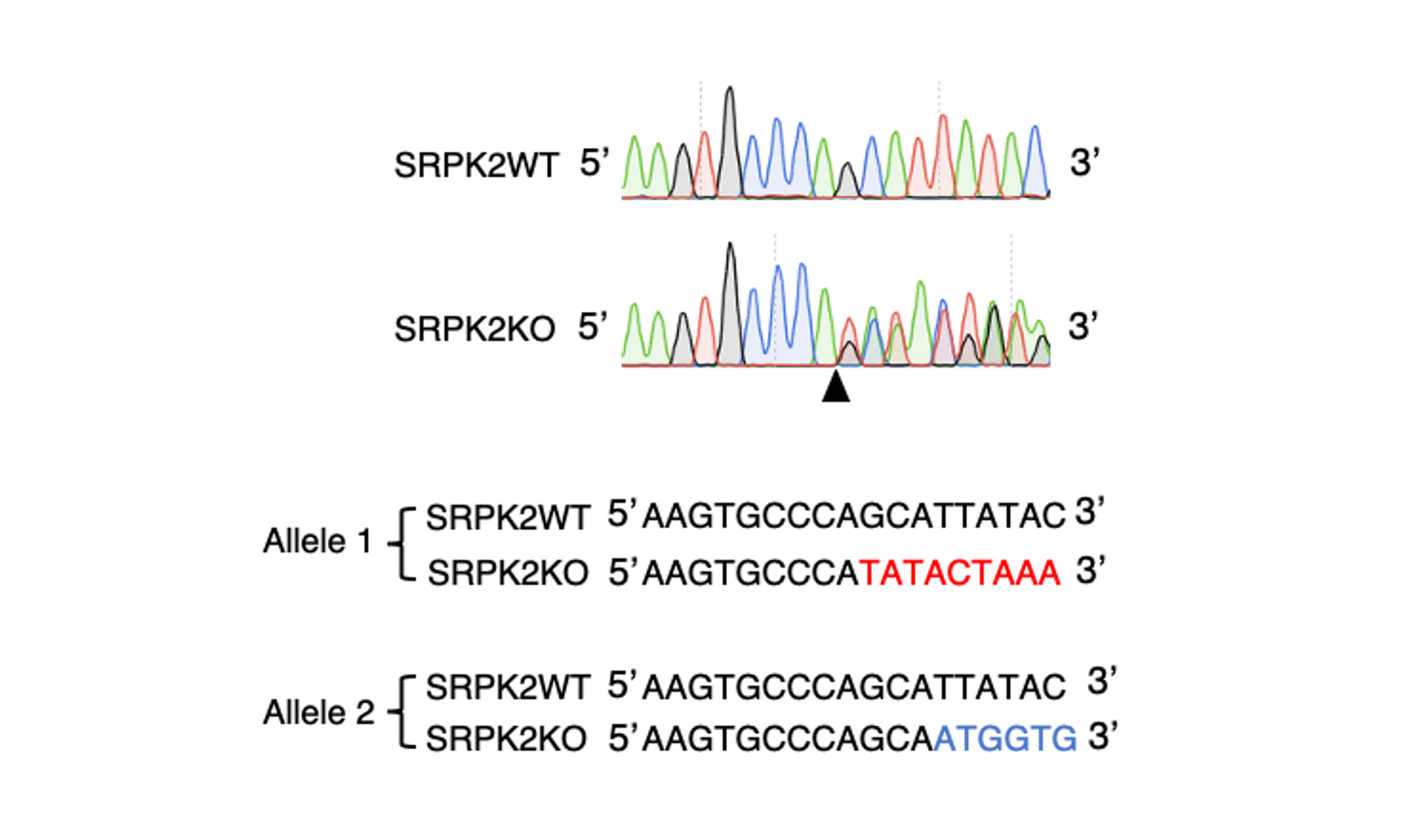

Supplement: S11 Fig — Genomic DNA from the cell lines was extracted for sequencing. The position where the DNA sequence of SRPK2 was altered by CRISPR-Cas9 is indicated (upper panels). Heterozygous sequencing results indicated that the knockout was biallelic and the altered DNA sequence in two alleles are labelled in red and blue respectively (bottom panels). (TIF) [file ppat.1011978.s011.tif]

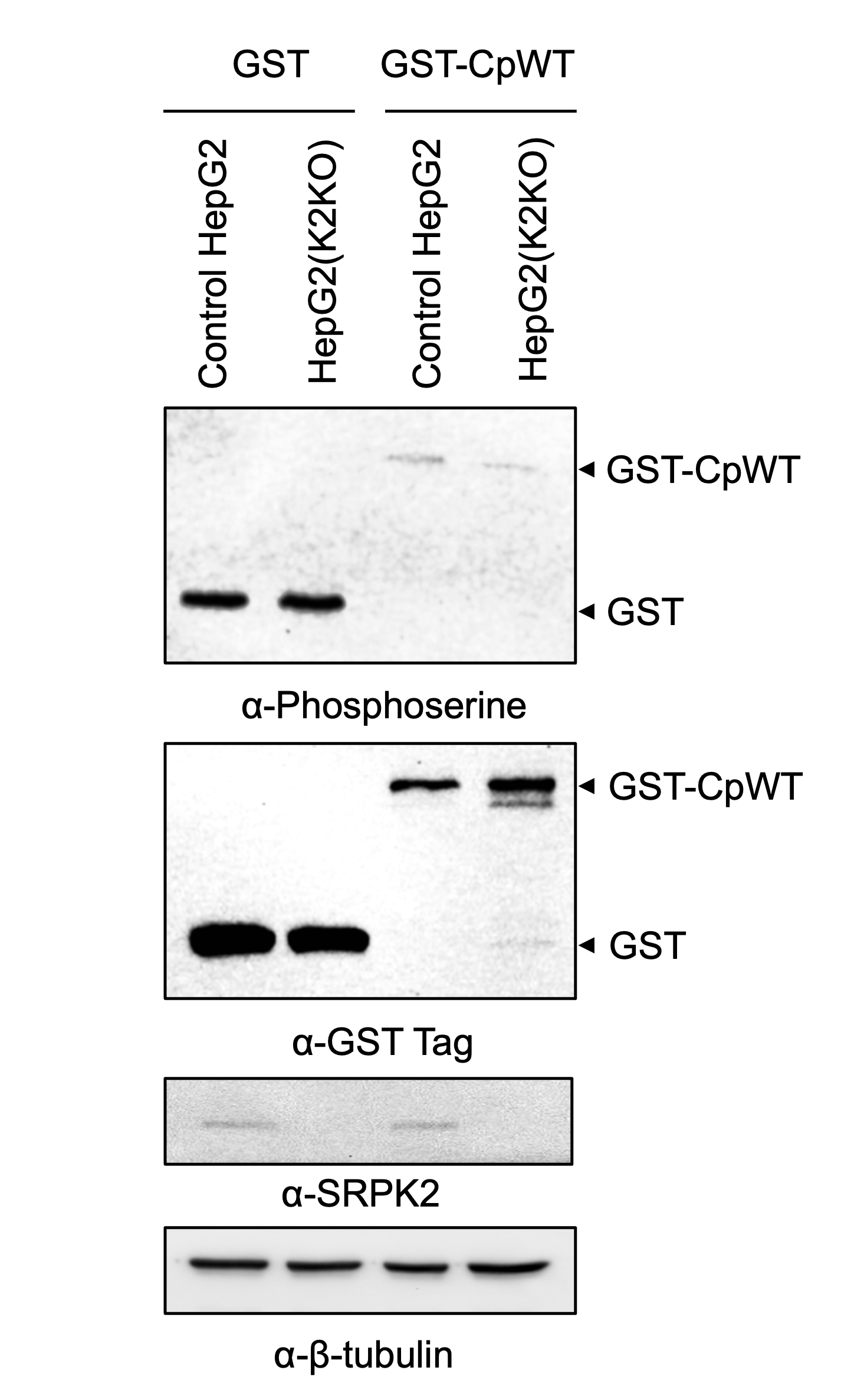

Supplement: S12 Fig — The effect of SRPK2 knockout on the phosphorylation of core protein in HepG2 and HepG2(K2KO) cell lines. Lysates of control HepG2 and HepG2(K2KO) cells were incubated with recombinant GST-CpWT. GST-CpWT was then isolated using glutathione resins and analyzed by western blot using anti-phosphoserine antibody. The phosphorylation of GST-CpWT using HepG2(K2KO) lysate was lowered when compared to that using control HepG2 cells. GST, which was phosphorylated by unknown kinase(s) in the lysates, was included as a control. (TIF) [file ppat.1011978.s012.tif]

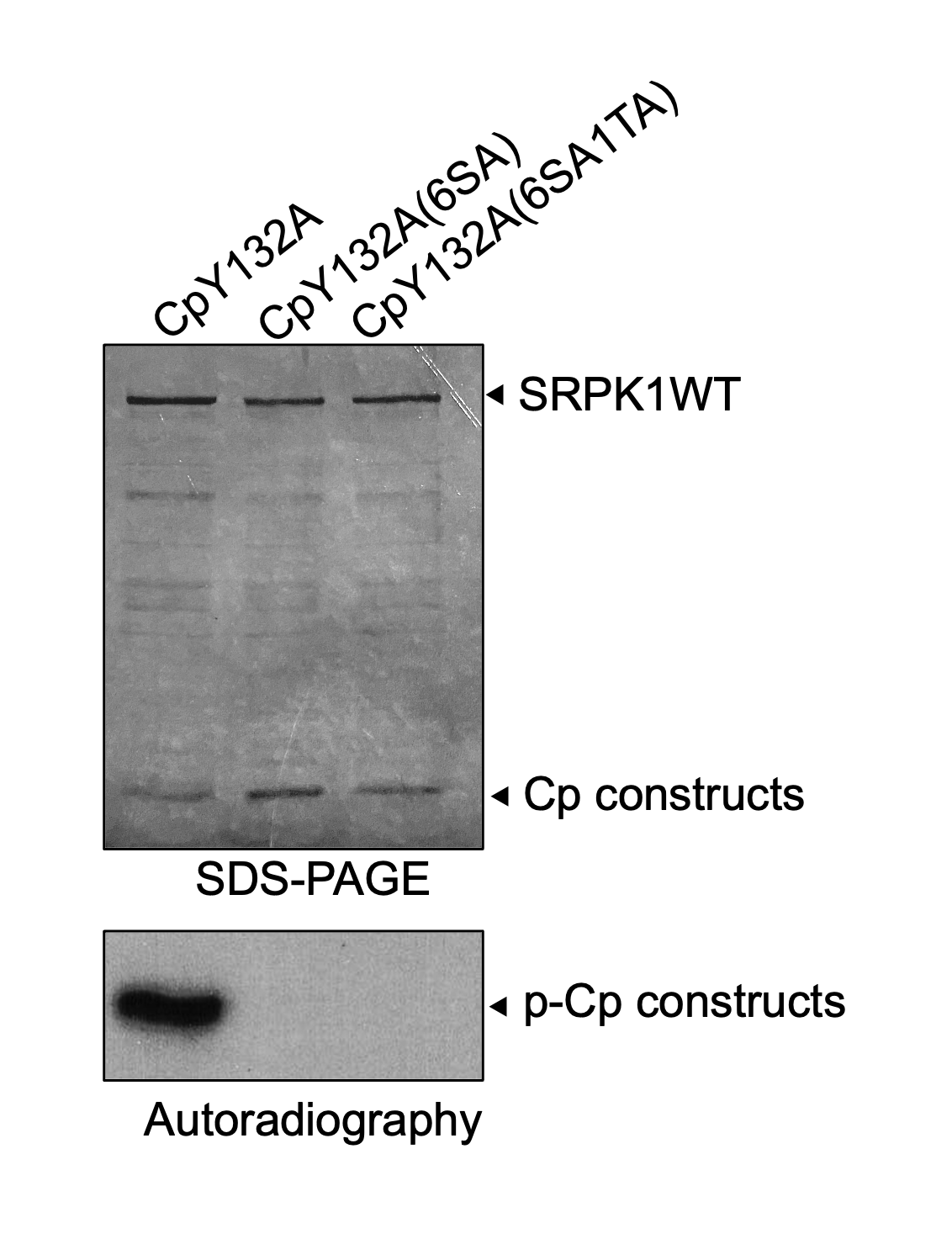

Supplement: S13 Fig — In vitro radioactive kinase assay was performed using SRPK1WT and the mutational Cp constructs, with six/seven alanine substitution at the phosphorylatable sites, in the presence of [32P]ATP. Reactions were quenched after 10 mins and analyzed by SDS-PAGE. The gel was visualized with Coomassie Blue staining and then subjected to autoradiography. Mutation of six serines to alanines abolished the phosphorylation of Cp by SRPK1. (TIF) [file ppat.1011978.s013.tif]
