# Supplementary material for: SRPK2 Mediates HBV Core Protein Phosphorylation and Capsid Assembly via Docking Interaction
Source: PLoS Pathog. 2024 Feb 7;20(2):e1011978. doi: 10.1371/journal.ppat.1011978 (PMC10878513; doi:10.1371/journal.ppat.1011978)
Supplement: S1 Table — (DOCX) [file ppat.1011978.s014.docx]

|  | HBV capsid  (EMD-37631) | SRPK2∆S1/HBV capsid complex  (EMD-38062) | Focus refined SRPK2∆S1  (EMD-37634) |
| --- | --- | --- | --- |
| Magnification | 150,000X | 150,000X | 150,000X |
| Voltage (kV) | 200 | 200 | 200 |
| Electron exposure (e^-^/Å^2^) | 50 | 50 | 50 |
| Defocus range (µm) | 0.8-2.2 | 0.5-3.0 | 0.5-3.0 |
| Pixel size (Å) | 1.938 | 1.938 | 1.938 |
| Initial particle images for 3D classification | 3,840 | 41,604 | 375,960 (after symmetry expansion) |
| Final particle images for 3D reconstruction | 1,773 | 6,266 | 58,259 |
| Box size (pixel) | 240 | 256 | 256 |
| Initial model used (EMDB code) | EMD3015 | EMD3015 | / |
| Symmetry imposed | I | I | C1 |
| Map resolution (Å) | 4.4 | 4.6 | 11 |
| FSC threshold | 0.143 | 0.143 | 0.143 |
| Map sharpening B-factor (Å^2^) | -91.493 | -127.457 | -396.178 |
| Micrographs | 270 | 824 | / |
| Particle extracted from micrographs | 6,042 | 54,774 | / |

**S1 Table. Data acquisition and processing of single-particle-analysis cryoEM**
